# AMBRA1 phosphorylation by CDK1 and PLK1 regulates mitotic spindle orientation

**DOI:** 10.1007/s00018-023-04878-6

**Published:** 2023-08-16

**Authors:** Fiorella Faienza, Federica Polverino, Girish Rajendraprasad, Giacomo Milletti, Zehan Hu, Barbara Colella, Deborah Gargano, Flavie Strappazzon, Salvatore Rizza, Mette Vixø Vistesen, Yonglun Luo, Manuela Antonioli, Valentina Cianfanelli, Caterina Ferraina, Gian Maria Fimia, Giuseppe Filomeni, Daniela De Zio, Joern Dengjel, Marin Barisic, Giulia Guarguaglini, Sabrina Di Bartolomeo, Francesco Cecconi

**Affiliations:** 1 Cell Stress and Survival Group, Center for Autophagy, Recycling and Disease (CARD), Danish Cancer Institute, Copenhagen, Denmark; 2 Department of Biology, University of Rome Tor Vergata, Rome, Italy; 3 Institute of Molecular Biology and Pathology, CNR National Research Council, Rome, Italy; 4 Cell Division and Cytoskeleton, Danish Cancer Institute, Copenhagen, Denmark; 5 Department of Pediatric Hemato-Oncology and Cell and Gene Therapy, Bambino Gesù Children’s Hospital, IRCCS, Rome, Italy; 6 Present Address: DNA Replication and Cancer Group, Danish Cancer Institute, 2100 Copenhagen, Denmark; 7 Department of Biology, University of Fribourg, Fribourg, Switzerland; 8 Department of Biosciences and Territory, University of Molise, Pesche, Italy; 9 IRCCS Fondazione Santa Lucia, Rome, Italy; 10 Physiopathologie et Génétique du Neurone et du Muscle, UMR5261, U1315, Institut NeuroMyogène, Univ Lyon, Univ Lyon 1, CNRS, INSERM, 69008 Lyon, France; 11 Redox Biology Group, Danish Cancer Institute, Copenhagen, Denmark; 12 Lars Bolund Institute of Regenerative Medicine and Qingdao-Europe Advanced Institute for Life Sciences, BGI Research, Shenzhen, China; 13 Department of Biomedicine, Aarhus University, Aarhus, Denmark; 14 National Institute for Infectious Diseases, IRCSS “L. Spallanzani”, Rome, Italy; 15 Department of Science, University “ROMA TRE”, 00146 Rome, Italy; 16 Present Address: Department of Woman and Child Health and Public Health, Gynecologic Oncology Unit, Fondazione Policlinico Universitario A. Gemelli IRCCS, Rome, Italy; 17 Department of Molecular Medicine, Sapienza University of Rome, Rome, Italy; 18 Center for Healthy Aging, University of Copenhagen, Copenhagen, Denmark; 19 Melanoma Research Team, Danish Cancer Institute, Copenhagen, Denmark; 20 Department of Drug Design and Pharmacology, University Of Copenhagen, Copenhagen, Denmark; 21 Department of Cellular and Molecular Medicine, Faculty of Health Sciences, University of Copenhagen, Copenhagen, Denmark; 22 Università Cattolica del Sacro Cuore and Fondazione Policlinico Universitario Agostino Gemelli IRCCS, Rome, Italy

**Keywords:** Phosphorylation, Cell cycle, Mitotic kinases, Mitotic spindle, NUMA1

## Abstract

AMBRA1 is a crucial factor for nervous system development, and its function has been mainly associated with autophagy. It has been also linked to cell proliferation control, through its ability to regulate c-Myc and D-type cyclins protein levels, thus regulating G1-S transition. However, it remains still unknown whether AMBRA1 is differentially regulated during the cell cycle, and if this pro-autophagy protein exerts a direct role in controlling mitosis too. Here we show that AMBRA1 is phosphorylated during mitosis on multiple sites by CDK1 and PLK1, two mitotic kinases. Moreover, we demonstrate that AMBRA1 phosphorylation at mitosis is required for a proper spindle function and orientation, driven by NUMA1 protein. Indeed, we show that the localization and/or dynamics of NUMA1 are strictly dependent on AMBRA1 presence, phosphorylation and binding ability. Since spindle orientation is critical for tissue morphogenesis and differentiation, our findings could account for an additional role of AMBRA1 in development and cancer ontogenesis.

**Supplementary Information:**

The online version contains supplementary material available at 10.1007/s00018-023-04878-6.

## Introduction

Autophagy and Beclin 1 regulator 1 (AMBRA1) has been initially discovered as a key factor for nervous system development, and its deficiency has been mainly associated with a defect of autophagy [1]. Since its discovery in 2007, AMBRA1 has emerged as a crucial signaling molecule in several signaling pathways ranging from autophagy, where AMBRA1 is an upstream positive regulator, to mitophagy, cell death and cell proliferation [2–8]. AMBRA1 is able to exert diverse cellular functions, thanks to its intrinsically disordered nature that confers to it scaffolding properties and the ability to interact with different molecular partners [9, 10]. Moreover, due to its plasticity, AMBRA1 is tightly regulated by post-translational modifications, such as activatory or inhibitory phosphorylations and degradative ubiquitylation [6, 11, 12]. AMBRA1 deficiency has been also associated with unbalanced cell proliferation and displacement of several regulators of differentiation during morphogenesis [1, 13] and the excessive proliferation rate of AMBRA1-deficient cells has been linked to enhanced susceptibility to form tumoral masses [2]. Indeed, in 2015, Cianfanelli et al. demonstrated that AMBRA1 is able to regulate cellular proliferation by promoting de-phosphorylation and degradation of the c-Myc proto-oncogene, thus linking AMBRA1 to cell cycle regulation [2]. Moreover, recently, an additional role of AMBRA1 in coordinating cell cycle progression and genomic stability through regulating CCND (CyclinD) stability has been elucidated [14]. AMBRA1, in fact, is part of the CRL4 ubiquitin ligase complex responsible for D-type cyclins degradation [15, 16].

A key step during cell cycle is cell division that is accomplished during M phase. Since any defects at this stage could cause genetic abnormalities in daughter cells, mitosis is a tightly regulated process, and several families of mitotic kinases ensure the fidelity of the entire process [17]. Among these, Cyclin dependent kinase 1 with its partner Cyclin B1 (hereafter referred as CDK1), is the major kinase and is essential for mitotic progression [18]. To successfully complete mitosis, CDK1 activity is adjuvated by Polo-like kinase (PLK1), another important mitotic kinase [19–21]. Indeed, CDK1 and PLK1 kinases collaborate for substrates phosphorylation on multiple sites, ensuring their proper spatio-temporal regulation [22–26].

Kinase activity is required for mitotic progression, which relies on the proper function of mitotic spindle machinery, that physically segregate chromosomes at opposite poles [17, 27]. In more detail, mitotic spindle is composed of microtubules, microtubule-associated proteins and motor proteins, and its stability and function is critical for cell division [27]. Spindle positioning is crucial for symmetric and asymmetric cell divisions, and establishes the position of the mitotic cleavage plane [28, 29]. Indeed, a conserved machinery for mitotic spindle positioning does exist and is composed by Leu-Gly-Asn repeat-enriched protein (LGN), Nuclear mitotic apparatus 1 (NUMA1) and Dynein/Dynactin complex [30–34]. The key molecule in the complex is NUMA1 that binds plasma membrane through LGN-Gα_i_ complex, and is able to bind both astral microtubules, to anchor mitotic spindle, and Dynein/Dynactin motor, to generate pulling-force and to position mitotic spindle [35–37]. NUMA1 is an essential protein for both spindle positioning and stability: in fact it localizes at the cell cortex, where it drives spindle orientation, and on mitotic spindle, where it promotes microtubules bundling to centrosomes [29, 38]. Interestingly, it has been demonstrated that NUMA1 mobilization from spindle poles to the cell cortex is regulated by several phosphorylation events mediated by different mitotic kinases such as CDK1, PLK1 and Aurora Kinase A (AURKA) [39–42].

In this work, we show that AMBRA1 is sequentially phosphorylated at mitosis by CDK1 and PLK1 on multiple sites. In particular, CDK1 is responsible for the early phosphorylations on T1209 and S1223, and it promotes additional late phosphorylation events by PLK1 on AMBRA1. Altogether, these phosphorylation events are critical for proper spindle function and orientation. Indeed, phosphorylated AMBRA1 can interact with NUMA1 and is responsible for NUMA1 proper localization at the cell cortex. Moreover, we observe that loss of AMBRA1 leads to PLK1 protein stabilization and to an increase in phospho-NUMA1 levels which, in turn, contributes to spindle orientation defects.

## Results

### AMBRA1 is phosphorylated during mitosis on T1209 and S1223

**Fig. 1:**
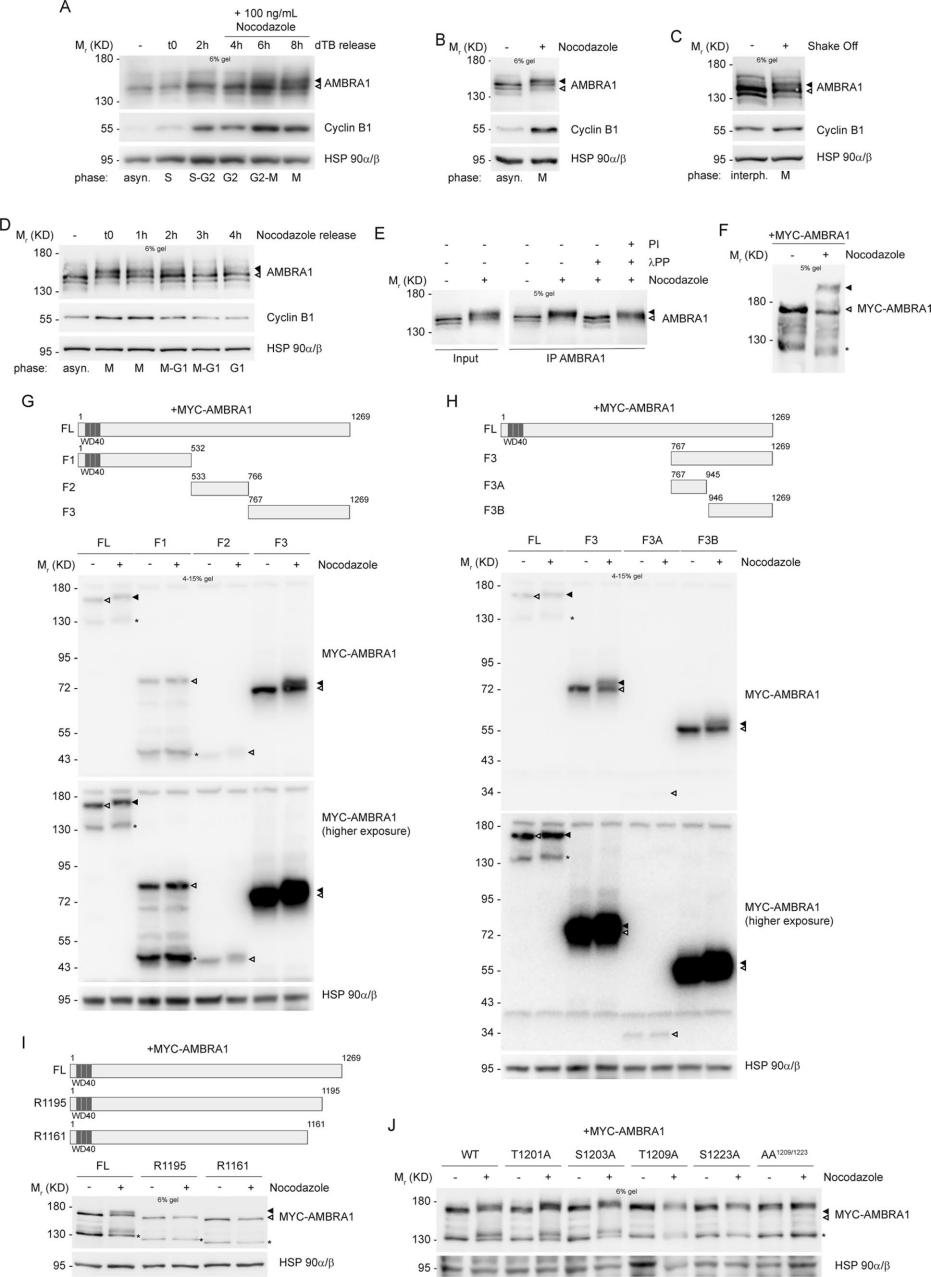
AMBRA1 is phosphorylated during mitosis at sites T1209 and S1223. **A**–**D** WB analysis of protein extracts from: **A** HeLa cells synchronized at G1/S boundary with a double Thymidine block (dTB), and released in the presence of 100 ng/mL Nocodazole, to arrest cells in mitosis; **B** HeLa cells synchronized at mitosis using 200 ng/mL Nocodazole; **C** HeLa cells in naturally-occurring mitosis after mitotic shake off; **D** HeLa cells treated with 200 ng/mL Nocodazole, and released. **E** HeLa cells synchronized at mitosis with Nocodazole, followed by AMBRA1 IP and in vitro phosphatase assay with *lambda* protein phosphatase (λPP) on immunoprecipitated protein; phosphatase inhibitors (PI) were used as a control. **F** HeLa cells transfected with MYC-AMBRA1 and then treated with Nocodazole. In this case protein extracts were separated by Phos-Tag SDS-PAGE and then analyzed by WB. **G–J** WB on protein extracts from: **G**, **H** HeLa cells transfected with MYC-AMBRA1 fragments and treated with Nocodazole. MYC-AMBRA1 is showed at a lower (up) and an higher (bottom) exposure. **I** HeLa cells transfected with MYC-AMBRA1 full-length (FL) or R1161 and R1195 truncated fragments, followed by treatment with Nocodazole. **J** HeLa cells transfected with MYC-AMBRA1 wild-type (WT) or phosphosilent (Alanine substitution) for T1201, S1203, T1209, S1223 and T1209/S1223 (AA^1209/1223^). Then cells were treated with Nocodazole. Cell cycle phases are specified for each lane in panels **A**, **B**, **C** and **D** (asyn. = “asynchronous”; interph. = “interphase”). Schematic representation of truncated fragments respect to full-length protein is provided above WB of panels **G**, **H** and **I**. (FL = “full-length”, F1 = “fragment 1”, F2 = “fragment 2”, F3 = “fragment 3”, F3A = “fragment 3A”, F3B = “fragment 3B”, WT = “wild-type”). WD40 domains (51–90, 93–133, 135–175) are highlighted in dark grey. The white arrow indicates AMBRA1 electrophoretic migration, while the black arrow indicates its mobility shift. AMBRA1 hypershift was visualized with low percentage acrylamide gels (5–6%) or with gradient pre-cast gel for blots in **G** and **H**. An asterisk marks a MYC-AMBRA1 degradation sub-product. Gel percentages are indicated in each WB panel

In order to analyze the expression pattern of AMBRA1 throughout the cell cycle, we decided to perform synchronization experiments in HeLa cells, by using some pharmacological and physical methods. By using Thymidine, we synchronized cells at the G1/S boundary, and then we released cells from the block, allowing them to go through S, G2 and M phases in a synchronous way. As shown in Fig. 1A and Fig. S1B, no differences in AMBRA1 protein levels were detected while an evident difference in AMBRA1 electrophoretic mobility was observed in M phase-entering cells, labelled by Cyclin B1 up-regulation. A similar electrophoretic mobility shift was observed in cells arrested at mitosis by using the microtubule-network destroying agent Nocodazole (Fig. 1B, Fig. S1D, S1E and S1F). We also observed an altered mobility shift of AMBRA1 in mitotic cells isolated by mitotic “shake off” from asynchronous populations (Fig. 1C). Moreover, the canonical AMBRA1 mobility pattern was soon restored by removal of the mitotic block and allowing cells to go through and over mitosis (as evident in Nocodazole-released cells) (Fig. 1D). The capability of the different pharmacological drugs to synchronize cells was monitored by Cyclin B1 protein level modulation and by FACS analysis (Fig. S1A, S1C and S1G). Next, in order to verify whether or not AMBRA1 hypershift was representative of a post-translational hyperphosphorylation, we subjected protein extracts from Nocodazole-treated cells to an in vitro phosphatase assay. As shown in Fig. 1E, the phosphatase was able to revert the protein mobility hypershift. Moreover, the Phos-Tag western blot of Nocodazole extracts further confirmed that AMBRA1 hypershift is due to phosphorylation (Fig. 1F), this indicating that AMBRA1 is phosphorylated during mitosis.

Next, in order to identify the AMBRA1 region modified by phosphorylation during mitosis we took advantage of AMBRA1 deletion constructs that encode for different fragments of the protein (Fig. 1G and H, schemes). The overexpression of these constructs in HeLa cells, followed by Nocodazole treatment, clearly demonstrates that the C-terminal part of AMBRA1 (fragment F3B, from aa 946 to 1269) is the region modified by the phosphorylation event, since its electrophoretic mobility is clearly modified by Nocodazole (Fig. 1G and H). To identify the specific amino acid residues modified by phosphorylation, we performed a mass spectrometry analysis on AMBRA1 immuno-purified from Asynchronous cells and from Nocodazole-treated cells. As shown in Table 1 and in Dataset 1, we identified several AMBRA1 “phosphosites”, with the majority of them mapping in the region corresponding to the F3B fragment. Then, to refine our analysis, we followed the AMBRA1 electrophoretic mobility pattern obtained by transfection of two additional deletion constructs, R1195 and R1161, in mitotic cells. In Fig. 1I, it is shown that neither R1195 nor R1161 can be phosphorylated in Nocodazole-treated cells, thus suggesting that the key residues for AMBRA1 mitotic phosphorylation map downstream of R1195. We thus generated phosphosilent mutants, by substituting Serine/Threonine with Alanine residues, for each site among those identified downstream of R1195, and then overexpressed those mutants (T1201A, S1203A, T1209A, S1223A) in HeLa cells. We analyzed the electrophoretic migration pattern of each mutant with respect to the wild-type (WT) protein, and found that T1201A and S1203A still show a mobility shift upon Nocodazole treatment, whereas T1209A and S1223A do not show an appreciable variation in the mobility pattern in the same conditions (Fig. 1J). Moreover, Alanine substitution of both T1209 and S1223 (AA^1209/1223^) completely abrogates the Nocodazole-induced mobility shift (Fig. 1J), this indicating the importance of these two residues for AMBRA1 phosphorylation at mitosis.

**Table 1:** Mascott Mass Spectrometry for phosphosites’ identification on AMBRA1 upon Nocodazole treatment

Peptide sequence	Modified amino acid residue	MASCOT score	Asynchronous	Nocodazole
S**S**ASPQEERTV	Ser637	40	–	+
LSSSA**S**PQEERT	Ser639	54	+	+
SQTGTEPGAAHTS**S**PQPS	Ser1176	37	+	+
LLPEAGQLAERGL**S**PRT	Ser1198	36	+	–
TSRGLLPEAGQLAERGLSPR**T**A	Thr1201	26	–	+
SWDQPG**T**PGREPTQPT	Thr1209	26	–	+
**S**WDQPGTPGREPTQPTLPSS**S**PVPIPV	Ser1203, Ser1223	25	–	+

^a^The modified residue is indicated in bold

^d^Here we show a selection of sites with a minimum MASCOT score of 25. The complete list of identified sites, with their relative MASCOT score is shown in Dataset 1

^c^Here we show sites with AMBRA1 isoform 3 numbering (Uniprot Q9C0C7), as in the whole text

### CDK1 phosphorylates AMBRA1

**Fig. 2:**
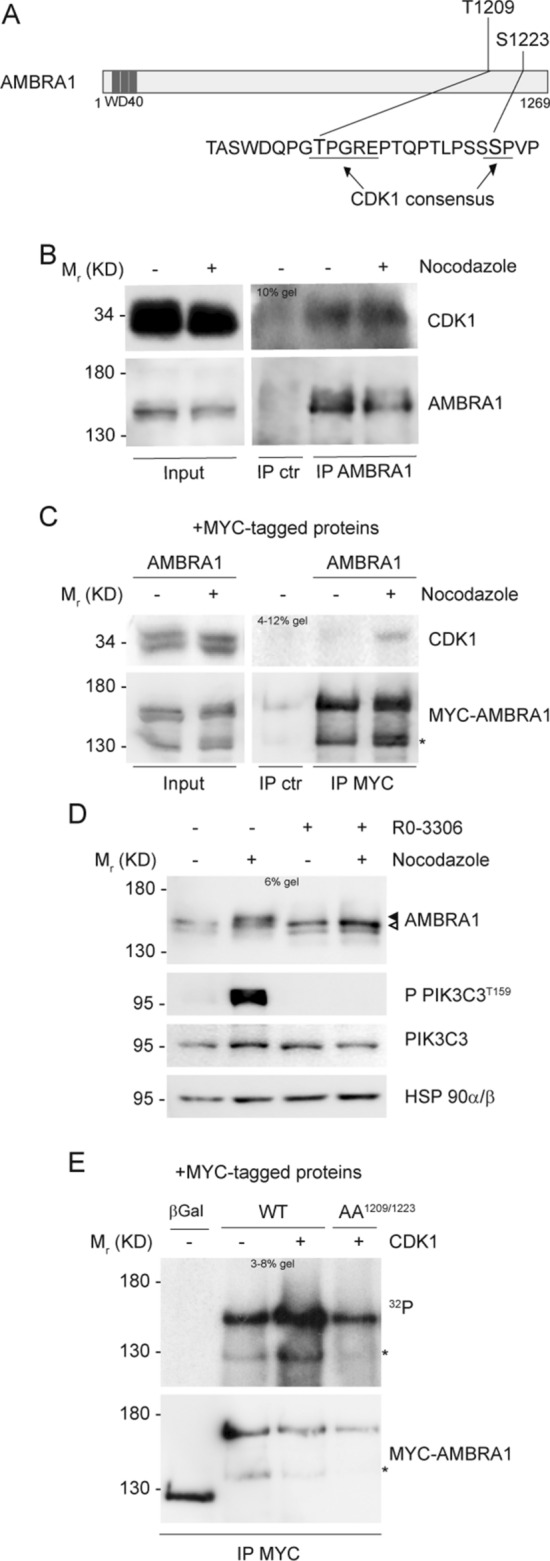
CDK1 phosphorylates AMBRA1 at T1209 and S1223. **A** Schematic representation of AMBRA1 protein, highlighting T1209 and S1223 with their respective surrounding sequence. CDK1 consensus is underlined. WD40 domains (51–90, 93–133, 135–175) are highlighted in dark grey. **B**, **C** WB of immunoprecipitated proteins following Nocodazole treatment, with mouse immunoglobulins used as control (IP ctr): **B** Endogenous proteins immunoprecipitated with anti-AMBRA1 antibody. **C** Overexpressed MYC-AMBRA1 immunoprecipitated using anti-MYC antibody. **D** WB of HeLa cells treated with Nocodazole and then with 9 μM RO-3306, in the last 10 min of treatment. The white arrow indicates AMBRA1 electrophoretic migration, while the black arrow indicates its mobility shift. AMBRA1 hypershift was visualized with a low percentage acrylamide gel (5–6%). **E** Autoradiography (^32^P signal) and WB of an in vitro kinase assay with recombinant CDK1/Cyclin B1 performed on MYC-AMBRA1 WT or AA^1209/1223^ and MYC-β-Galactosidase (βGal) as control. Proteins were immunoprecipitated using anti-MYC antibody following HeLa cells transfection with the relative constructs. An asterisk marks a MYC-AMBRA1 degradation sub-product. Gel percentages are indicated in each WB panel

In order to identify a candidate kinase for AMBRA1 phosphorylation at mitosis, we used online available bioinformatics tools, as Scansite (http://scansite.mit.edu/) and ELM (http://elm.eu.org/), in addition to literature data [43]. Matching all this information we speculated that CDK1, the major mitotic kinase, could be a potential kinase for both T1209 and S1223 phosphorylation (Fig. 2A). To test this hypothesis, we first checked whether the two proteins could interact upon Nocodazole treatment and, indeed, we found that they co-immunoprecipitated in both endogenous and overexpression conditions (Fig. 2B and C, and Fig. S2A). Then, to test if AMBRA1 was phosphorylated by CDK1, we used RO-3306, following Nocodazole treatment, to inhibit CDK1 activity in mitosis. RO-3306 treatment was able to reduce AMBRA1 phosphorylation, as well as CDK1-mediated Phosphatidylinositol 3-kinase catalytic subunit type 3 (PIK3C3) phosphorylations [44] (Fig. 2D). Notably, despite CDK1 inhibition, cells were synchronized at mitosis, as monitored by phospho-Ser10 H3 staining (Fig. S2B). Finally, we performed an in vitro kinase assay, using ^32^P radioisotope-labeled ATP, on immunoprecipitated WT and AA^1209/1223^ AMBRA1, using the recombinant CDK1/Cyclin B1 complex. As shown in Fig. 2E, CDK1 is able to phosphorylate WT AMBRA1, but not the double phosphosilent mutant AA^1209/1223^. Notably, AMBRA1 exhibits a basal phosphorylation in the absence of CDK1 that is most likely due to the activity of kinases co-precipitated with AMBRA1, e.g. the Unc-51 like kinase-1 (ULK1) [12]. Altogether, these findings strongly suggest that CDK1 is the kinase responsible for AMBRA1 phosphorylation on T1209 and S1223 at mitosis.

### CDK1-mediated phosphorylation primes PLK1 phosphorylation on AMBRA1

**Fig. 3:**
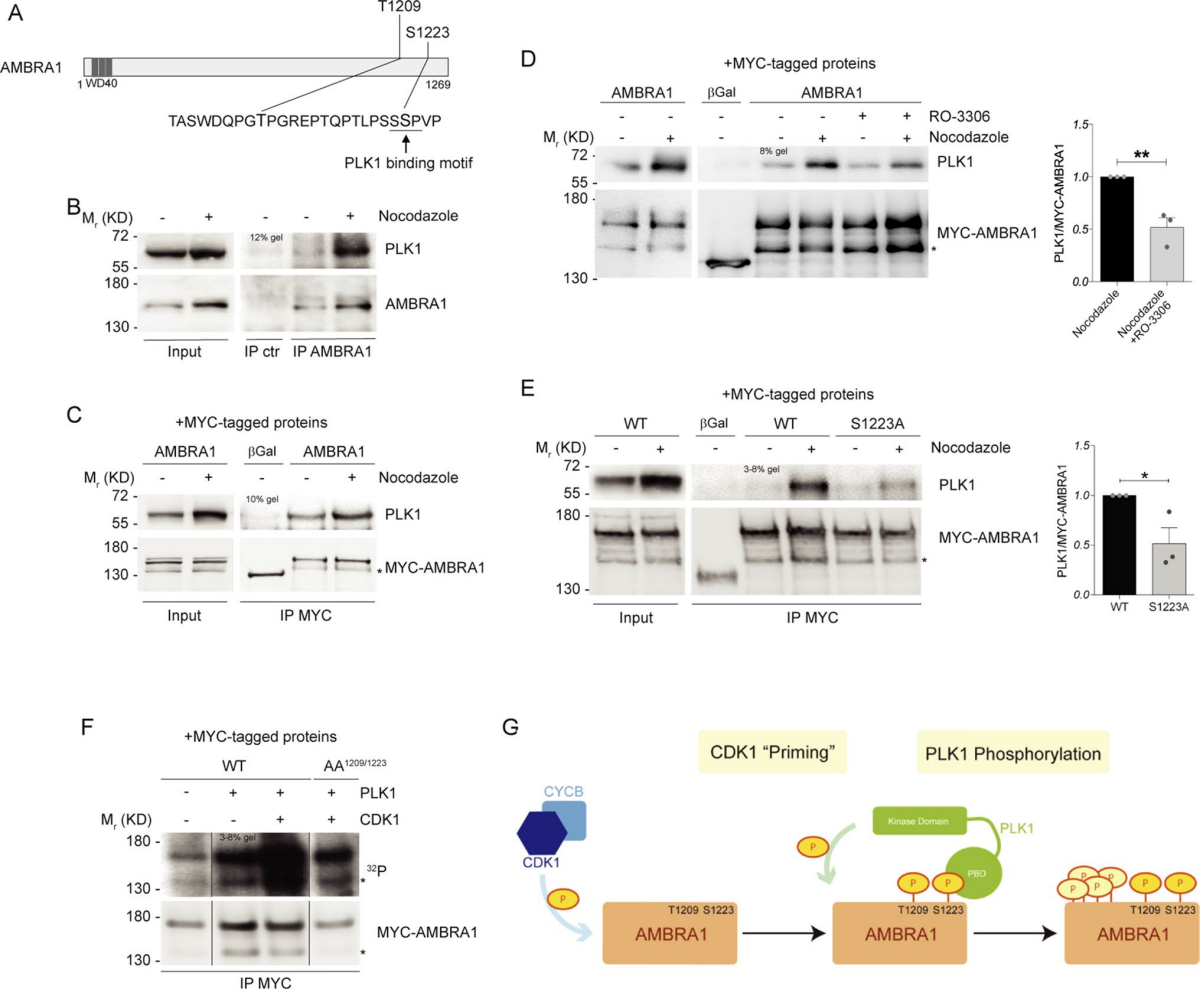
CDK1-mediated phosphorylation primes PLK1 phosphorylation on AMBRA1. **A** Schematic representation of AMBRA1 protein, highlighting T1209 and S1223 with their respective surrounding sequence. PLK1 binding motif is underlined. WD40 domains (51–90, 93–133, 135–175) are highlighted in dark grey. **B–E** WB of immunoprecipitated proteins following Nocodazole treatment: **B** Endogenous proteins were immunoprecipitated with AMBRA1 antibody. Mouse immunoglobulins were used as control (IP ctr). **C–E** Overexpressed MYC-AMBRA1 and MYC-β-Galactosidase as control immunoprecipitated using anti-MYC antibody. **D** HeLa cells treated in the last 10 min of Nocodazole with 9 μM RO-3306. **E** HeLa cells transfected with MYC-AMBRA1 WT or S1223 phosphosilent mutant (S1223A, Alanine substitution). Quantification, only for mitotic protein extracts, as mean ± s.e.m. of three independent experiments is shown, and significance is **(p < 0.005) for **D** and *(p < 0.05) for **E,** by Student’s T test. **F** Autoradiography (^32^P signal) and WB of an in vitro kinase assay with recombinant CDK1/Cyclin B1 and PLK1 performed on MYC-AMBRA1 WT or AA^1209/1223^ and MYC-β-Galactosidase (βGal) as control. Proteins were immunoprecipitated using anti-MYC antibody following HeLa cells transfection with the relative constructs. The image represented here derives from the same experiment in which lanes in the middle were cropped. **G** Model for AMBRA1 phosphorylation at mitosis: AMBRA1 is at first phosphorylated by CDK1/Cyclin B1 on T1209 and S1223, then pS1223 is bound by PLK1 that further phosphorylate AMBRA1 on additional sites. An asterisk marks a MYC-AMBRA1 degradation sub-product. Gel percentages are indicated in each WB panel

Mitotic entry is marked by a peak of protein phosphorylation that is driven by several mitotic kinases [17]. Among others, Polo-like kinase 1 (PLK1) is able to phosphorylate its substrates after a priming phosphorylation by CDK1 [20, 21, 45]. Given the evident AMBRA1 hypershift in mitotic cells, we hypothesized that phospho-AMBRA1 could bind and be then phosphorylated by PLK1, too. In order to assess whether PLK1 could bind AMBRA1, we performed a bioinformatic analysis of the AMBRA1 sequence, by using GPS-Polo software (http://gps.biocuckoo.org/), and we found several potential sites for PLK1 binding. Among these sites, the CDK1-phosphorylated S1223 was predicted to be bound by the PLK1 substrate binding domain (Polo Box Domain, PBD), and its surrounding sequence is also in line with the motif identified by Elia et al. [45] (Fig. 3A). Next, to test if PLK1 could bind AMBRA1 after CDK1 phosphorylation, we performed co-immunoprecipitation experiments in control and Nocodazole-treated cells. We found that AMBRA1 and PLK1 are able to co-immunoprecipitate both in endogenous and in overexpression conditions (Fig. 3B and C, and Fig. S2C). As expected, the interaction is phosphorylation-dependent, since it can be prevented by treating cells with RO-3306 (a CDK1 inhibitor, Fig. 3D). Hence, we tested the ability of AMBRA1 S1223 phosphosilent mutant to bind PLK1, and we found that this mutant has a reduced capability to bind the kinase, when compared with WT AMBRA1, upon Nocodazole treatment (Fig. 3E). Moreover, we found that the double phosphosilent mutant (AA^1209/1223^), has also a reduced ability to bind PLK1 (Fig. S2D). These results demonstrate that PLK1 is able to bind phospho-AMBRA1 through its PBD domain, and that phospho-S1223 is a key residue, but most likely not the only one, for this interaction.

Next, to confirm that PLK1 can phosphorylate AMBRA1 after CDK1 “priming” phosphorylation, we performed a combined in vitro kinase assay. Indeed, we performed the kinase assay in two consecutive rounds: in the first round we subjected AMBRA1 to a kinase reaction with CDK1 (as in Fig. 2E), whilst in the second one we exposed AMBRA1 to PLK1. By using this approach, a significant phosphorylation of WT AMBRA1 but not of its AA^1209/1223^ mutant was observed (Fig. 3F, S2E plus Datasets 2A and 2B and S2F). Taken together, these results support a specific program of phosphorylation, in which AMBRA1 is at first phosphorylated by CDK1 on T1209 and S1223, and then by PLK1 on additional sites, at mitosis (Fig. 3G).

### Lack of AMBRA1 phosphorylation causes several mitotic defects

**Fig. 4:**
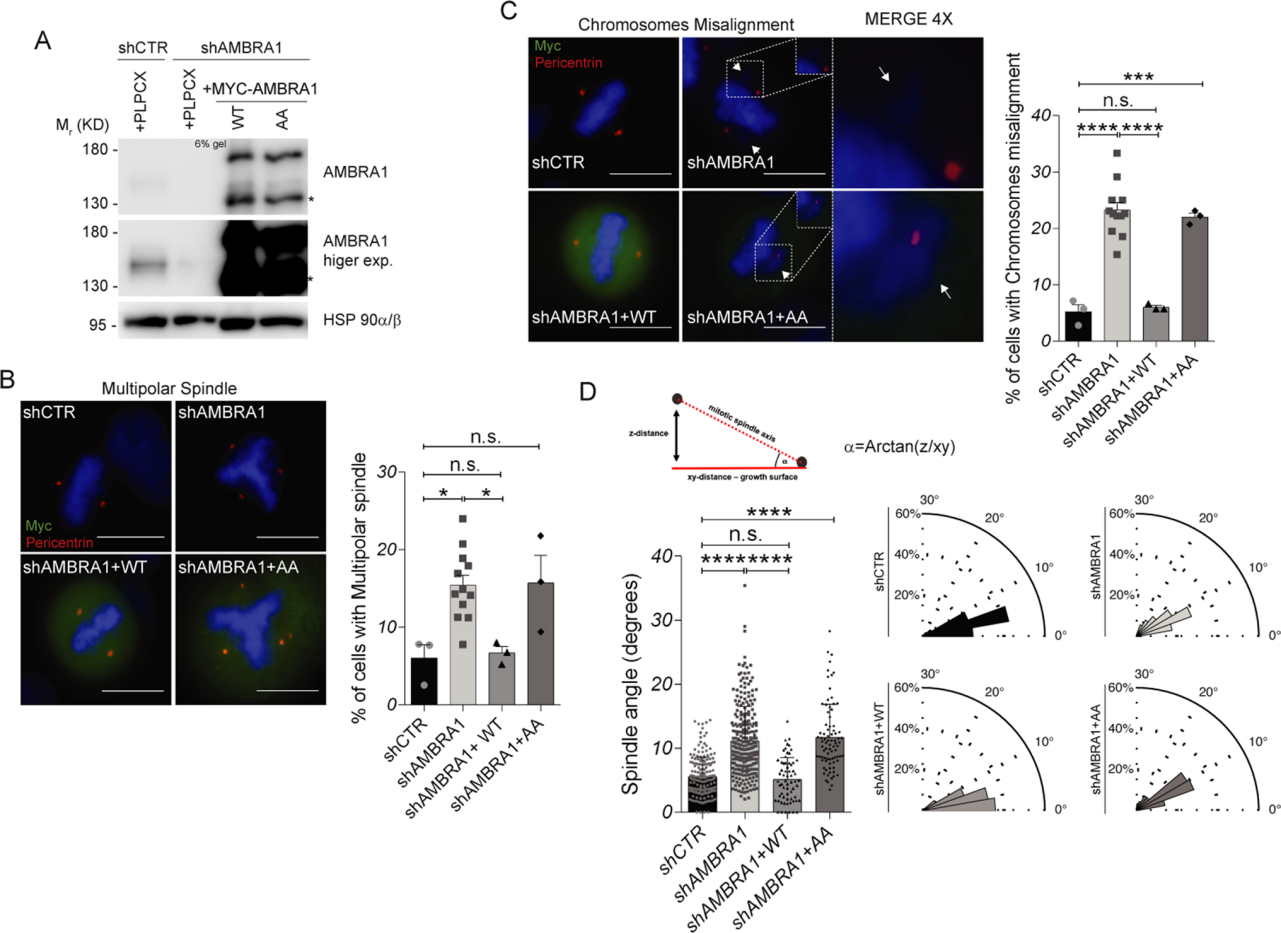
Lack of AMBRA1 phosphorylation causes mitotic defects. **A** WB of stably *AMBRA1*-silenced HeLa cells transfected with WT and phosphosilent (AA^1209/1223^) MYC-AMBRA1 or with the PLPCX empty vector. An asterisk marks a MYC-AMBRA1 degradation sub-product. Gel percentage is indicated in WB panel. **B**, **C** The same cells as in **A** were grown on coverslips and stained with anti-MYC antibody, to identify transfected cells, and anti-Pericentrin antibody, to identify centrosomes. Nuclei were stained with DAPI (scale bar = 8 μm). Merged images are shown, with 4X magnification shown only in **C**. White arrows indicate the misaligned chromosomes. The percentage of cells with each defect is represented in the graphs on the right. Bars show mean ± s.e.m. of the percentage of cells which exhibits the indicated defect, and significance is calculated with ordinary one-way ANOVA: * = p < 0.05; *** = p < 0.001; **** = p < 0.0001; n.s. = p > 0.05. Analysis was performed on 100–150 cells for each experiment. **D** The same cells as in **A** were grown on coverslips and stained with anti-MYC antibody, to identify transfected cells, and anti-Pericentrin antibody, to identify centrosomes and build the cell division axis. Nuclei were stained with DAPI. On the top left is shown a scheme of how mitotic spindle angle (α) is calculated. On the bottom left, mitotic spindle angle measure (degrees) is shown for all conditions. Bars show mean ± s.e.m. of 50–250 measures, and significance is **** (p < 0.0001) by ordinary one-way ANOVA On the bottom right mitotic spindle angle measure (degrees) is shown as a polar distribution

In the attempt to uncover AMBRA1 phosphorylation function, we reconstituted *AMBRA1*-silenced cells with WT and AA^1209/1223^ MYC-AMBRA1 (Fig. 4A), in search of mitotic defects. As shown in Fig. 4B and C, both *AMBRA1*-deficient and AA^1209/1223^-rescued cells exhibit an increase in the percent of cells with multipolar spindle (Fig. 4B) and chromosomes misalignment (Fig. 4C). Importantly, WT AMBRA1-rescued cells behave as control cells, as shown in Fig. 4B and C. Next, in the same system, we analyzed mitotic spindle orientation. Indeed, mitotic spindle orientation is evaluated in cultured cells through the measure of mitotic spindle angle in metaphase, which is increased in case of misorientation. As shown in Fig. 4D, *AMBRA1*-deficient and AA^1209/1223^-rescued cells show a strong and significant increase in the mitotic spindle angle, which is fully rescued by WT AMBRA1, thus suggesting that AMBRA1 and its phosphorylation have an important effect on mitotic spindle orientation. Similar results were obtained in *AMBRA1* silenced HCT-116 cells (Fig. S3A and S3B) and in *AMBRA1* knock-out (KO) 2FTGH (Fig. S3C and S3D) and HeLa cells generated with the CRISPR/Cas9 technology (Fig. S4A), in which we observed an increase in the number of cells showing a multipolar spindle (Fig. S4B) and chromosomes misalignment (Fig. S4C), together with a significant increase in mitotic spindle angle (Fig. S4D). Notably, also in these cells, WT AMBRA1 overexpression rescues the normal spindle angle value, while the phosphosilent mutant AA^1209/1223^ is unable to achieve a similar rescue (Fig. S4D). Moreover, AA^1209/1223^ MYC-AMBRA1 overexpression in control cells does not affect the spindle angle. This, indeed, suggests that the phosphosilent mutant does not exert a dominant negative effect (Fig. S4D). In addition, the role of AMBRA1 in spindle orientation was further confirmed by spinning-disk confocal live cell imaging of *AMBRA1* KO cells (Fig. S4E) and *AMBRA1* RNAi-treated HeLa cells (Fig. S4G and S4H), showing that metaphase spindles were tilted under these conditions (Fig. S4E and S4H). Of note, no difference was detected in metaphase spindle length of *AMBRA1* KO cells (15.89 ± 0.86 in control compared to 15.54 ± 1.15 in *AMBRA1* KO cells) (Fig. S4F). On the other hand, AMBRA1 phosphorylation had no effect on autophagy regulation during mitosis, as evidenced by ATG14 Ser29 phosphorylation (Fig. S5A) and LC3 II accumulation (Fig. S5B). Altogether, these data support the hypothesis that AMBRA1 phosphorylation is important for mitotic spindle function, independently from its role on autophagy.

### AMBRA1 phosphorylation regulates mitotic spindle orientation through NUMA1

**Fig. 5:**
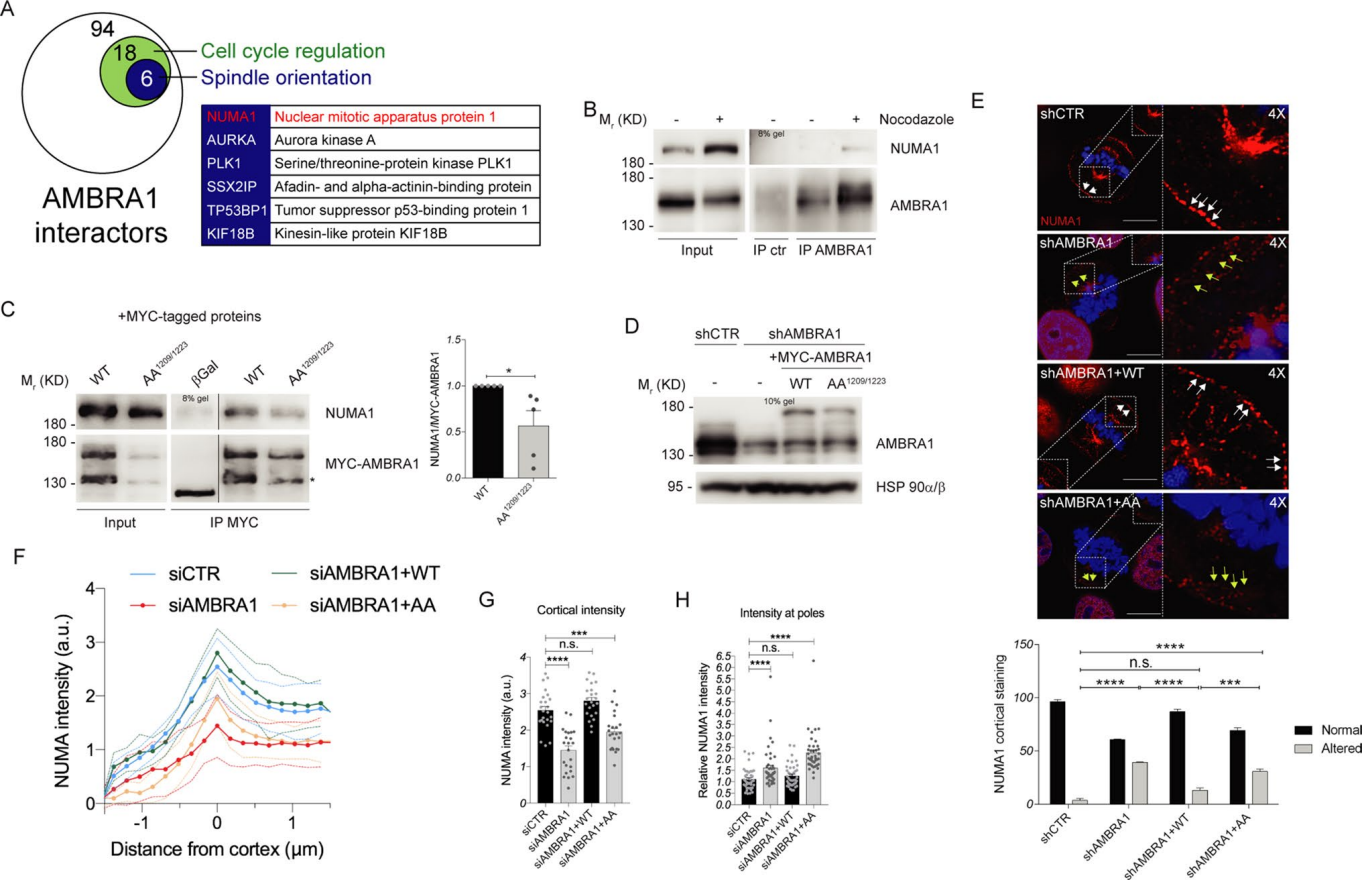
AMBRA1 phosphorylation regulates mitotic spindle function through NUMA1 mislocalization from cortex. **A** Diagram showing significant changing interaction for AMBRA1 upon Nocodazole treatment. HeLa cells, stably transfected with FLAG-AMBRA1, were grown in light (Lys^0^–Arg^0^) and heavy (Lys^6^–Arg^10^) SILAC medium. FLAG-AMBRA1 was immunoprecipitated and then eluates were analyzed by mass spectrometry. Here cell cycle regulators are indicated in the green circle while proteins involved in spindle orientation are indicated in the blue circle and specified in the flanking table. NUMA1 is highlighted in red. **B**, **C** WB of immunoprecipitated proteins following Nocodazole treatment: **B** Endogenous proteins were immunoprecipitated with AMBRA1 antibody. Mouse immunoglobulins were used as control (IP ctr). **C** Overexpressed MYC-AMBRA1 WT or AA^1209/1223^ and MYC-β-Galactosidase as control immunoprecipitated using anti-MYC antibody. The image derives from the same experiment in which lanes in the middle were cropped. Quantification, only for mitotic protein extracts, as the mean ± s.e.m. of five independent experiments is shown on the right, and significance is * (p < 0.05) by Student’s T test. **D** WB of stably *AMBRA1*-silenced or transfected with WT and phosphosilent (AA^1209/1223^) MYC-AMBRA1 HeLa cells. **E** Analysis of NUMA1 staining for the same cells in **D**. Merged images of a single confocal plane and 4X magnification are shown for each condition (scale bar = 10 μm). White and yellow arrows indicate NUMA1 cortical staining. The number of cells with an altered NUMA1 staining at the cortex is shown in the graph on the bottom, and significance is n.s. (p > 0.05), *** (p < 0.001) or **** (p < 0.0001) by two-way ANOVA. **F** NUMA1 line profiles across the cortex from HeLa cells stably expressing GFP-NUMA1 transfected with indicated siRNAs and mCherry-PLPCX or mCherry-AMBRA1 WT or AA^1209/1223^. Solid line represents the mean intensity with s.d. represented by dotted lines. N(number of cells, number of independent experiments): siCTR + mCherry (24, 3); si*AMBRA1* + mCherry (23, 3); si*AMBRA1* + mCherry-AMBRA1 WT (23, 3); si*AMBRA1* + mCherry-AMBRA1 AA^1209/1223^ (23, 3). **G** NUMA1 levels at the cortex extracted from (**F**). Individual cortical intensities with mean ± s.e.m. are plotted. P-values were calculated using Student’s T test and significance is: n.s. (p > 0.05), *** (p < 0.001), **** (p < 0.0001). **H** The same cells as in (**F**) were used for quantification of NUMA1 intensity at the poles of mitotic spindles at metaphase. Levels at individual poles are plotted along with mean ± s.e.m. N(number of cells, number of independent experiments): siCTR + mCherry (21, 3); si*AMBRA1* + mCherry (23, 3); si*AMBRA1* + mCherry-AMBRA1 WT (21, 3); si*AMBRA1* + mCherry-AMBRA1 AA^1209/1223^ (20, 3). P-values were calculated using Mann–Whitney U test and significance is: n.s. (p > 0.05), **** (p < 0.0001). An asterisk marks a MYC-AMBRA1 degradation sub-product. Gel percentages are indicated in each WB panel

To identify the molecular partners of AMBRA1 in mitotic spindle regulation, we next took advantage of a Stable isotope labeling with amino acids in cell culture (SILAC) mass spectrometry analysis, performed on AMBRA1-overexpressing cells, comparing an asynchronous population with the mitotic-enriched one. Among the proteins whose interaction with AMBRA1 was significantly increased in Nocodazole-treated cells, mass spectrometry analysis suggested some crucial players for mitotic spindle progression, such as NUMA1, AURKA and PLK1 (Fig. 5A). Among these, NUMA1 is a spindle-positioning regulator, whose proper localization and function are required for correct orientation [29]. We confirmed AMBRA1-NUMA1 interaction, in mitotic cells, by co-immunoprecipitation experiments performed in asynchronous and in Nocodazole-treated cells (Fig. 5B). Moreover, the interaction was significantly reduced in cells overexpressing AA^1209/1223^ AMBRA1 mutant (Fig. 5C). Notably, we found that *AMBRA1*-deficient cells show a significant de-localization of NUMA1 from the cell cortex that is recovered by WT AMBRA1, but not by AA^1209/1223^-AMBRA1 (Fig. 5D and E). Remarkably, NUMA1 mislocalization from the cortex was also evident by live cell imaging of GFP-NUMA1 HeLa cells depleted for *AMBRA1* or rescued with AA^1209/1223^-AMBRA1 (Fig. 5F and G, as well as in *AMBRA1* KO HeLa cells (Fig. S6A–C). Interestingly, NUMA1 de-localization from cortex goes along with NUMA1 accumulation at spindle poles (Fig. 5H and Fig. S6D), while no differences in NUMA1 protein levels were detected (Fig. S7A). Additionally, despite AMBRA1 ability to interact with NUMA1 we could not observe any difference in localization between WT and AA^1209/1223^ AMBRA1 (Fig. S6E plus Movie 1 and Movie 2).

**Fig. 6:**
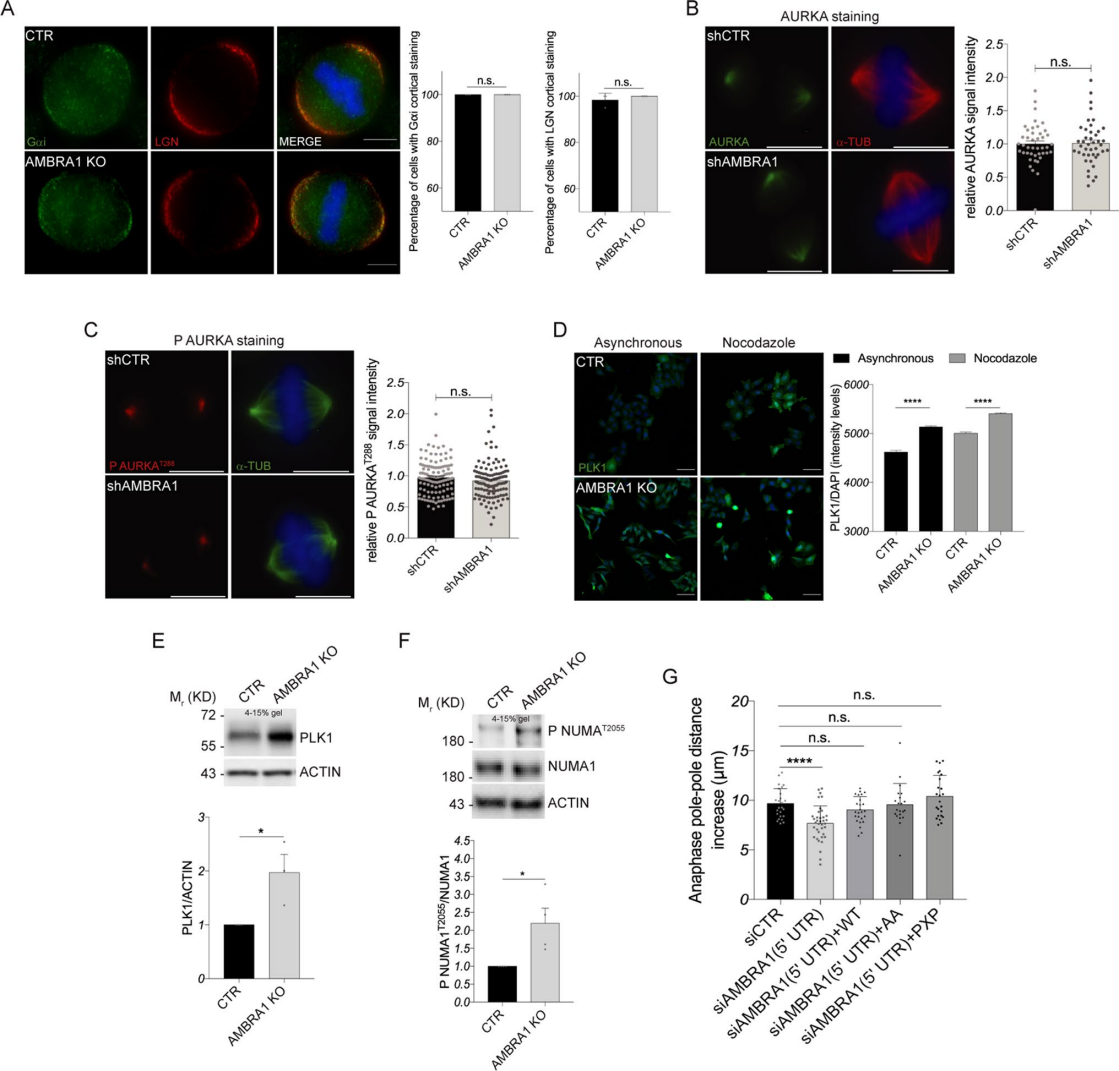
AMBRA1 depletion impairs PLK1 and CDK1 signaling on NUMA1. **A** Analysis of LGN and Gαi staining for CTR and *AMBRA1* KO HeLa cells. Fluorescent microscopy images are shown on the left (scale bar = 8 μm), while on the right are shown the relative graphs as mean ± s.e.m. of three independent experiments. Significance is n.s. (p > 0.05) by Student’s T Test. **B**, **C** AURKA (**B**) and P AURKA^T288^ (**C**) staining of stable *AMBRA1*-silenced HeLa cells. The spindle is marked with α-TUBULIN antibody. Fluorescent microscopy images are shown on the left (scale bar = 8 μm), while on the right is shown the relative signal intensity as mean ± s.e.m. of about 40 measures for AURKA (**B**) and of about 120 measures for P AURKA^T288^ (**C**). Significance is n.s. (p > 0.05) by Student’s *T* Test. **D** High-content imaging analysis of PLK1 staining in CTR and *AMBRA1* KO HeLa cells treated or not with Nocodazole. Nuclei were stained with DAPI (scale bar = 150 μm). PLK1 signal intensity is shown in the graph on the right. Bars show mean ± s.e.m. of 2000–10,000 measures, and significance is ****(p < 0.0001) by ordinary one-way ANOVA. **E**, **F** WB analysis of protein extracts from CTR and *AMBRA1* KO HeLa cells treated with Nocodazole. Quantification, as mean ± s.e.m. three (**E**) and four (**F**) independent experiments, is shown at the bottom, and significance is * (p < 0.05) (**E**) by Student’s T test. **G** Quantification of pole-pole distance increase over metaphase spindle length during anaphase. Individual values with mean ± s.d. are plotted. N(number of cells, number of independent experiments): siCTR (25, 3); si*AMBRA1* (5’ UTR) (37, 3); si*AMBRA1* (5’ UTR) + WT (25, 3); si*AMBRA1* (5’ UTR) + AA (21, 3); si*AMBRA1* (5’ UTR) + PXP (24, 3). Significance is n.s. (p > 0.05) and **** (p < 0.0001) by Student’s T Test. Gel percentages are indicated in each WB panel

In order to explore the molecular mechanism, by which AMBRA1 regulates NUMA1 localization, we first analyzed the expression of NUMA1 upstream regulators LGN and Gαi, responsible for NUMA1 anchorage at the plasma membrane [28]. As shown in Fig. 6A and Fig. S7B, C, we did not observe any difference in their protein levels nor localization. Therefore, we decided to investigate the involvement of the kinases AURKA, PLK1 and CDK1, which are all known to regulate NUMA1 localization through phosphorylation events and which are all AMBRA1 interactors (see Figs. 2B, 3B, 5A and Fig. S7E). We observed that the expression and localization of both total and active phospho- AURKA were unchanged in the presence or in the absence of AMBRA1 (Fig. 6B, C and Fig. S7D). Furthermore, co-immunoprecipitation of the AMBRA1 mutant AA^1209/1223^ with AURKA was not significantly changed when compared with the WT protein (Fig. S7E). This implies that the phosphorylation state of AMBRA1 affects its capability to bind NUMA1, but not AURKA.

Conversely, when we analyzed PLK1 expression and localization, we observed an increase of its protein levels in *AMBRA1* KO asynchronous and mitotic cells, due to an increased protein stability (Fig. 6D, E and Fig. S7F). PLK1 protein increase in *AMBRA1* KO cells suggests, indeed, enhanced activity in phosphorylating its substrates, most likely in concert with CDK1. Although we could not detect an increased kinase activity of PLK1 on NUMA1, due to the unavailability of any specific tools, we could analyze the CDK1-mediated phosphorylation of NUMA1 by taking advantage of a specific anti-phospho-T2055 antibody. As shown in Fig. 6F, we observed an increased expression of the CDK1-phosphorylated form of NUMA1 in *AMBRA1*-depleted cells. It is known that NUMA1 phosphorylation at Thr2055 by CDK1 is required for spindle pole association of NUMA1 at the onset of mitosis and that its dephosphorylation by the phosphatase PP2A leads to an enhancement of NUMA1 at the cell cortex in anaphase [41]. Due to AMBRA1 capability to interact with both CDK1 and PP2A [2], we hypothesized that AMBRA1 could regulate NUMA1 localization and activity through its phospho/dephosphorylation state. However, the expression of AMBRA1 AA^1209/1223^ or PP2A (PXP) mutant, unable to bind PP2A, in siAMBRA1 cells (Fig. S7G and S7H) was not able to reproduce the anaphase defect induced by AMBRA1 depletion (Fig. 6G), this suggesting that AMBRA1 regulates NUMA1 through additional unknown (phosphorylation) mechanism/s.

## Discussion

Since its discovery in 2007, AMBRA1 has been mainly associated with autophagy and several efforts have been done to dissect its role at a molecular level [2, 4, 6–8, 10–12, 46–51]. In 2015, we associated an unbalanced cell proliferation observed in *AMBRA1*-deficient cells with tumorigenesis, based on AMBRA1 ability to interact with the Protein Phosphatase 2A (PP2A) and to promote c-Myc proto-oncogene de-phosphorylation and proteasomal degradation [2]. Recently, we strengthened this finding with the discovery of AMBRA1 ability to also regulate D-type cyclin protein levels through the CLR4-DDB1 complex [14, 15]. Altogether, these findings link AMBRA1 to cell cycle regulation and open new perspectives on its network of regulation. For this reason, we decided to analyze AMBRA1 expression profile during cell cycle and found that AMBRA1 exhibits a remarkable change in its electrophoretic mobility during mitosis, with this suggesting a post-translational modification with potential interesting functions. Indeed, here we show that a significant AMBRA1 hypershift is due to protein phosphorylation on multiple sites, located in the C-terminal region of AMBRA1. Of note, AMBRA1 is an intrinsically-disordered protein and it is known to be phosphorylated on multiple sites, whose roles are still in part unknown [52]. Several post-translational modifications have been proven to dynamically regulate AMBRA1, both in basal conditions and in response to diverse stimuli [6, 9, 12], and are probably responsible for conformational changes that trigger AMBRA1 interactions with different molecular partners.

By biochemical and molecular approaches, we thus identified T1209 and S1223 as key residues for AMBRA1 phosphorylation at mitosis. As these two residues lie in a CDK1-consensus motif, we investigated the involvement of this kinase in AMBRA1 phosphorylation, and we were able to demonstrate that AMBRA1 is a substrate of CDK1. Moreover, we found that phospho-AMBRA1 is recognized and further phosphorylated by PLK1, another kinase playing a key role in mitotic entry, progression and exit [17, 19, 21]. Indeed, we found not only that AMBRA1 and PLK1 interact during mitosis, but also that their interaction relies on AMBRA1 phosphorylation by CDK1. Altogether, these findings allow us to propose a model for AMBRA1 phosphorylation at mitosis, in which AMBRA1 is initially phosphorylated by CDK1 on T1209 and S1223, to create a pS1223-PLK1 binding site. Then, PLK1 recognizes pS1223 and further phosphorylates AMBRA1, similarly to what previously described for other proteins regulated by these two kinases (Fig. 3G) [22–26].

In recent years, autophagy regulation at mitosis has been debated with different studies supporting the hypothesis that autophagy could be inhibited or induced during this cell cycle phase [44, 53–57]. Intriguingly, beside the debated bulk autophagy regulation, it has been proven that selective forms of autophagy, such as doryphagy, assist mitosis progression ensuring fidelity of chromosomes segregation [58–61]. Since AMBRA1 is mainly involved in autophagy regulation, it is conceivable that its phosphorylation status at mitosis is necessary for a fine-tuning of autophagy regulation during this phase of the cell cycle. However, our data clearly show that AMBRA1 phosphorylation at mitosis is not related to autophagy regulation, in line with other autophagy factors that show autophagy-independent functions during mitosis [62–64]. Instead, we found that AMBRA1 phosphorylation is mainly associated with the mitotic spindle function and orientation, since its deficiency or phosphorylation defects cause alterations in bipolar spindle organization, chromosomes alignment and spindle positioning.

To add molecular insights to our findings, we decided to identify by mass spectrometry AMBRA1 dynamic interactions at mitosis. By using this approach, we observed a significant increase in AMBRA1 interaction with cell cycle-related proteins, and intriguingly with several proteins involved in spindle orientation. Among these, NUMA1 protein caught our attention for its key role in mitotic spindle function; NUMA1 localizes at the mitotic spindle, where it drives microtubule bundling to centrosomes, and at the cell cortex, where it is responsible for mitotic spindle anchoring and proper positioning, together with the Dynein/Dynactin complex [29, 35–37]. Strikingly, we found that AMBRA1 interaction with NUMA1 depends on AMBRA1 phosphorylation. Moreover, AMBRA1 phosphorylation is required for a proper cortex localization of NUMA1, which is in turn responsible for proper spindle orientation. It is well established that NUMA1, Gαi and LGN act as a core protein complex that recruits Dynein/Dynactin to the cell cortex [30, 36, 65]. This, in turn, generates pulling forces and orients and/or positions the mitotic spindle [31, 66, 67]. However, AMBRA1 does not affect Gαi and LGN localization or protein levels, suggesting that its function in this context is mostly related to NUMA1 localization.

Given the role of AMBRA1 as a scaffold protein [9, 10], it is plausible that the localization and dynamics of AMBRA1 and NUMA1 are most likely dependent on the ability of AMBRA1 to keep proteins in the right conformation and interact with each other. Given that CDK1, PLK1 and AURKA phosphorylate NUMA1, regulating its cortical localization, it is conceivable that AMBRA1 is involved in the regulation of these phosphorylations. Interestingly, we observed that AMBRA1 is able to interact with all these kinases, having different consequences. AURKA is able to phosphorylate NUMA1 on different sites, inducing its mobilization from the spindle pole toward the cortex [39]. Given that AMBRA1 loss or lack of phosphorylation causes a decrease in NUMA1 cortical fraction, it would be possible in principle that AMBRA1 could favor NUMA1 phosphorylation by AURKA. However, we found that AMBRA1 interaction with AURKA is not phosphorylation-dependent and has no effect on AURKA localization, protein levels and activation status, this excluding AURKA from the molecular mechanism of AMBRA1-dependent regulation of spindle orientation. PLK1 and CDK1 also phosphorylate NUMA1 to regulate its localization, but their phosphorylations have an opposite effect with respect to AURKA, causing NUMA1 accumulation at spindle poles [41, 42, 68]. In line with this, AMBRA1 could antagonize these phosphorylations or favors dephosphorylation. Interestingly, the CDK1 phosphorylated T2055 on NUMA1 is dephosphorylated by PP2A [41, 68] and we demonstrated in 2015 that AMBRA1 is able to promote PP2A phosphatase activity on c-Myc [2]. Strikingly, we found that AMBRA1 regulates PLK1 stability, and that its loss causes an increase in PLK1 protein levels. PLK1 stability is controlled by APC/C and SKP1/CUL1/SCF ubiquitin ligases [69] and AMBRA1 is known to regulate cullin E3 ligases [11, 14, 15]. In this scenario, AMBRA1 might control PLK1 protein levels being part of a cullin-based E3 ligase complex. Increased PLK1 protein levels have been linked to an increased chromosomal instability, due to its increased kinase activity [70], suggesting that overabundant PLK1 in *AMBRA1* KO cells may result in an increased PLK1 phosphorylation of NUMA1 and consequent spindle pole retention. Moreover, we found that AMBRA1 loss also causes an increase in T2055 phosphorylation, which is not dependent on AMBRA1-PP2A interaction. In sum, our findings suggest that AMBRA1 impacts NUMA1 phosphorylation status, with consequences on its localization and spindle orientation. Last, AMBRA1 effect on NUMA1 localization most likely affects also NUMA1 function at spindle poles, as demonstrated by the occurrence of multipolar spindles and chromosome misalignments in the absence of AMBRA1 and its phosphorylation. Indeed, NUMA1 accumulation at spindle poles has been associated with centrosome clustering-dependent multipolarity in cancer cells with supernumerary centrosomes [71]. Although we could not assess by which pathway AMBRA1 regulates NUMA1 localization, we identified NUMA1 phosphorylation status as the key factor, influenced by AMBRA1, being responsible for the observed phenotype. The strong interconnection between CDK1 and PLK1 activity, together with their effect on the same AMBRA1 target, suggest a mechanism by which AMBRA1 is first phosphorylated by CDK1 and PLK1 to interact with NUMA1 and then regulates NUMA1 phosphorylation status, most likely keeping it in a dynamic complex with kinases.

In sum, our results disclose a new autophagy-independent role for AMBRA1 in controlling spindle orientation during mitosis. Mitotic spindle orientation is critical for the proper morphogenesis of epithelial tissues, since it determines cells positioning within the tissue, as well as differentiation factors segregation at mitosis [28, 37, 72]. Indeed, defects in spindle orientation have been linked to unbalance in stem cell pool maintenance and may give rise to several consequences, ranging from the impairment of embryonic development to an enhanced susceptibility to develop cancer [28, 72, 73]. Based on this evidence, we can thus speculate that displacement of patterning regulators, unbalanced cell proliferation, and tumor susceptibility (all been observed in AMBRA1-deficient models), are due, at least in part, to the role of AMBRA1 in mitotic spindle orientation.

## Materials and methods

### Cell cultures

HeLa, Hek293, HCT-116 and 2FTGH cells were cultured in DMEM (Lonza) in presence of 10% FBS (Gibco Life Technologies) and 1% Penicillin/Streptomycin mix (Gibco Life Technologies). Cells were grown at 37 °C and 5% CO_2_ in a humidified atmosphere.

For transfections, cells were incubated with plasmids and Lipofectamine 2000 (Thermo Fisher Scientific) in Opti-MEM medium (Gibco Life Technologies) for 4–6 h and then were cultured in DMEM for 24 or 48 h. For RNA interference (RNAi) experiments, cells were transfected at 30–50% confluence using Lipofectamine RNAiMAX (Thermo Fisher Scientific) for 48 h or 72 h.

For CDK1 kinase activity inhibition, cells were treated with Nocodazole (see cell synchronization methods section) overnight and then were treated with 9 μM RO-3306 (Sigma-Aldrich). For protein synthesis and degradation inhibition, cells were treated with 100 μg/mL cycloheximide (Sigma-Aldrich) or 5 μM MG132 (Sigma-Aldrich).

### Stable cell lines generation

For stable *AMBRA1* RNA interference, a lentiviral pLK0.1 plasmid targeting *AMBRA1* mRNA in 3′UTR was used (Sigma-Aldrich, TRCN0000168652 clone). The lentiviral production was obtained by co-transfecting pMDLg and psPAX2 plasmids in HEK293T cells and supernatants were collected 48 h post-transfection. Stable *AMBRA1* downregulated cells were obtained by infecting twice (18 h and 4 h) and selecting them by adding Puromycin (Sigma-Aldrich) to a final concentration of 2.5 μg/μL. As control, a pLK0.1 plasmid that does not target any known mammalian gene was used.

For stable FLAG-AMBRA1 transfection we used cells available in our laboratory, while for stable MYC-AMBRA1 WT and AA^1209/1223^-reconstituted cell lines we used *AMBRA1*-downregulated cells. Viruses were produced as previously described [6].

HeLa GFP-NUMA1 cells were generated by GFP-NUMA1 plasmid transfection and subsequent selection with 600 μg/mL of Hygromycin B (Gibco Life Technologies). GFP-positive cells were enriched using BD FACSAria (BD Biosciences) cell sorter.

### CRISPR editing of AMBRA1

Single-guide RNAs (sgRNAs) targeting exon 4 of human *AMBRA1* were designed using the public available tool gRNA-identifier ZiFiT Targeter [74] and screened for off-target events using the Cas-OFFinder software [75]: *AMBRA1* sgRNA #1, 5′-GAACCATAATATCTATATTA-3′; *AMBRA1* sgRNA #2, 5′-GCTGCCTAGATGGGGAGGTT-3′. The sgRNAs were cloned into the U6-driven Cas9 expression vector (pSpCas9(BB)-2APuro; 48,319, Addgene). CRISPR/Cas9 *AMBRA1* KO clones of HeLa cells were generated by the Dual-fluorescent surrogate system as described in Zhou et al. [76]. For 2FTGH *AMBRA1* KO cells were generated using CRISPR-Cas9 lentiviral vectors specific for *AMBRA1* (Hs*AMBRA1* sgRNA, K0079606, K0079608, ABMGood) and control sgRNA (Scramble sgRNA, K010, ABMGood). Transfected cells were selected with Puromycin (Sigma-Aldrich) and used as an heterogenous mixed population without clonal selection.

### Cell synchronization methods

For double Thymidine block and release, cells were treated twice with 2 mM Thymidine (Sigma-Aldrich) for 16–18 h, with a release of 8–9 h between the two treatments, and then were released. 4 h post-release, cells reaching M-phase were blocked adding 100 ng/mL Nocodazole (Sigma-Aldrich) to the medium. Cells were also directly synchronized at mitosis using either 200 ng/mL Nocodazole or 100 μM Monastrol (Sigma-Aldrich) for 16–18 h. Naturally occurring mitosis were collected with mitotic shake off as previously described [77].

### Plasmids and mutagenesis

MYC-β-Galactosidase, FLAG-AMBRA1, and MYC-AMBRA1 WT were cloned in a modified pLPCX vector (Clonetech), as previously described [1]. AMBRA1 deletion constructs encoding for fragments (F1, F2, F3, F3A and F3B) were previously generated and described [1]. AMBRA1 deletion fragments truncated at R1195 and R1161 were generated by PCR (primers: Fw. 5′-ATGAAGGTTGTCCCAGAAAAGAATGCTG-3′, Rev. R1195 5′-GCTCTGCCAGTTGCCCGGCCTCT-3′ Rev. R1161 5′-GCGGTGAATGCGGTGGCTGACGATG-3′), and subsequent cloning into PLPCX-MYC vector. AMBRA1 phosphosilent mutants were generated by site-directed mutagenesis of WT MYC-AMBRA1 with PCR (primers: T1201A 5′-TAAGCCCCCGGGCAGCTTCCTGGGACCAGCC-3′, S1203A 5′-GCCCCCGGACAGCTGCCTGGGACCAGCCTGG-3′, T1209A 5′-CCTGGGACCAGCCTGGGGCCCCTGGGCGGGAGCCAA-3′, S1223A 5′-CCCTGCCCTCTTCCGCCCCTGTCCCCATTCC-3′). Double phosphosilent mutant for T1209 and S1223 (AA^1209/1223^), was generated as described above, using as template MYC-AMBRA1 S1223A and primers for T1209 Alanine substitution (same primer as above for T1209A). mCherry-AMBRA1 AA^1209/1223^ and PXP were produced by subcloning AMBRA1 AA^1209/1223^ and PXP into mCherry-pLPCX vector. mCherry-PLPCX and mCherry-AMBRA1 WT were produced in the lab as previously described [1]. GFP-NUMA1 plasmid was a kind gift from prof. M. Mapelli (IEO, European Institute of Oncology, Milan, Italy) [39].

### siRNA for transient RNA interference

Transient RNA interference were achieved using non-targeting control siRNA (D-001810-01-05, Dharmacon Inc.) (sequence: 5′-UGGUUUACAUGUCGACUAA-3′), and custom-designed si*AMBRA1* (sequence: 5′-GGCCUAUGGUACUAACAAA-3′) or siAMBRA1 (5’UTR) (sequence: 5′-GGA CAA CUU ACA AGG ACC-3′).

### SILAC mass spectrometry

For SILAC mass spectrometry, stably FLAG-AMBRA1 transfected cells were cultured in SILAC medium (Silantes) composed of DMEM without stable Glutamine and without Arginine and Lysine, supplemented with 10% FBS, 2 mM l-stable Glutamine, 0.398 mM l-Arginine unlabeled (Arg^0^) or ^13^C^15^N labeled (Arg^6^), and 0.798 mM l-Lysine unlabeled (Lys^0^) or ^13^C labeled (Lys^10^). Unlabeled cells (Arg^0^–Lys^0^) were DMSO treated as control, while labeled cells (Arg^6^–Lys^10^) were treated with Nocodazole, as described in cell synchronization methods section, to have a mitotic population of cells. Protein extracts were then immunoprecipitated, as described in immunoprecipitation section, and analyzed by mass spectrometry, as described in mass spectrometry analysis section.

### Immunoprecipitation

Immunoprecipitation was performed on HeLa protein extracts using either HEMG buffer (25 mM Hepes pH 8.0, 12.5 mM MgCl_2_, 0.1 mM EDTA pH 8.0, 10% Glycerol, 100 mM NaCl, 0.5% Triton X-100) or CHAPS buffer (40 mM Hepes pH 7.4, 10 mM β-glycerophosphate, 0.3% CHAPS, 150 mM NaCl). Lysis was performed with selected buffer plus protease inhibitors (Protease Inhibitors Cocktail, Sigma-Aldrich) and phosphatases inhibitors (1 mM Sodium fluoride and 1 mM Sodium orthovanadate, Sigma-Aldrich). After quantification with DC protein assay (BIO-RAD), 1–2 mg of protein extracts were incubated with 1–2 μg of primary antibodies for MYC (tag), AMBRA1, CDK1, or PLK1 (see antibodies section) overnight with rotation at 4 °C. After antibodies binding, immuno-complexes were purified with 20 μL of Protein-G sepharose (Roche), for monoclonal primary antibodies, or Protein-A sepharose (Roche), for polyclonal primary antibodies, for 1 h with rotation at 4 °C. Purified complexes were washed 4–5 times with the buffer with 5 min centrifugation at 500* g*, and samples were used for kinase assay or for western blot (WB) analysis (see WB section). Equal amounts of total proteins were used for every experiment. Where a graph with quantification is present the amount of prey protein is always normalized over the amount of bait protein immunoprecipitated.

For mass spectrometry Tandem affinity purification (TAP) buffer (10 mM Tris–HCl pH 8.0, 150 mM NaCl, 10% Glycerol, 0.5% NP-40) was used. Cells transfected with FLAG-AMBRA1 WT or F3 were lysed with TAP buffer plus protease and phosphatase inhibitors as above, plus 1 mM PMSF (Sigma-Aldrich). Between 6 and 8 mg of proteins were immunoprecipitated with 300 μL of anti-FLAG M2 affinity gel (Sigma-Aldrich) for 3 h with rotation at 4 °C. After binding, immune-complexes were washed 4–5 times with TAP buffer with 5 min centrifugation at 500* g* and then eluted in two rounds with 200 ng/μL FLAG-peptide (Sigma-Aldrich). Samples were then subjected to mass spectrometry analysis for phosphosites or significant changing interaction detection.

### Western blot

Cells were lysed with CelLytic M (Sigma-Aldrich) or RIPA buffer plus protease and phosphatase inhibitors, as described in immunoprecipitation section, and cellular debris was removed by 15 min centrifugation at 10,000* g*. Protein extracts, obtained by cell lysis or immunoprecipitation, were denatured with Laemmli Sample Buffer 4X (LSB, NuPAGE-Novex-invitrogen Life technologies) plus 12% β-mercaptoethanol and with boiling of samples for 10 min at 95 °C. Denatured samples were then subjected to SDS-PAGE electrophoresis on acrylamide gel (BIO-RAD) and subsequent electro blotted to a Nitrocellulose (Amersham biosciences) or Polyvinylidene difluoride (PVDF, Millipore) membrane. Then, the membrane was blocked with 5% nonfat dry milk or BSA (Sigma-Aldrich), and was incubated with primary antibodies overnight, with rotation at 4 °C. Detection was possible thanks to horseradish peroxidase-conjugated secondary antibodies. Chemiluminescent reaction was induced with ECL plus (Millipore) and images were acquired with a digital camera (Fluor Chem SP, Alpha Innotec; Amersham Imager 600, Amersham). Background adjustment and cropping of images were done with ImageJ and Photoshop softwares.

### Phos-Tag SDS-PAGE

Phos-Tag Acrylamide gel was realized adding to resolving gel 50 μM Phos-Tag™ AAL (FUJIFILM Wako Chemicals U.S.A. Corporation) and 10 mM MnCl_2_. Then Phos-Tag gel was subjected to conventional SDS-PAGE. Prior to electro blotting the gel was washed twice in 1 mM EDTA dissolved in water and then in 1 mM EDTA dissolved in Blotting Buffer. Then WB was performed as described in the WB section.

### Antibodies

The primary antibodies that were used are: mouse anti-AMBRA1 antibody (Santa Cruz Biotechnology); mouse anti-Cyclin B1 antibody (Santa Cruz Biotechnology); rabbit anti-HSP 90 α/β antibody (Santa Cruz Biotechnology); rabbit anti-ACTIN antibody (Sigma-Aldrich); mouse anti-MYC(tag) antibody (Santa Cruz Biotechnology); rabbit anti-CDK1 antibody (Santa Cruz Biotechnology); rabbit anti-P Thr159 PIK3C3 antibody (kindly provided by prof. Yuan [44]); rabbit anti-PIK3C3 (Cell Signaling Technology); mouse anti-PLK1 antibody (Millipore); rabbit anti-NUMA1 antibody (Abcam); rabbit anti-P Thr2055 NUMA1 antibody (kindly provided by prof. Gönczy [41]); mouse anti-AURKA antibody (BD Transduction Laboratories); mouse rabbit-P Thr288 AURKA (Cell Signaling Technology); rabbit anti-Pericentrin antibody (Abcam), mouse anti-α-Tubulin (Sigma-Aldrich); rabbit anti-HSP-90 α/β antibody (Cell Signaling); mouse anti-HSP-90 α/β antibody (Enzo Life Sciences); rabbit anti-Phospho Serine (Millipore); rabbit anti-Phospho Threonine (Millipore); mouse anti-Gαi (Santa Cruz Biotechnology); mouse anti-LGN (kindly provided by prof. M. Mapelli, for WB); rabbit anti-LGN (Sigma-Aldrich, for IF); mouse anti-P Ser10 H3 (Millipore). For WB detection, horseradish peroxidase-conjugated goat anti-rabbit and goat anti-mouse antibodies (BIO-RAD) were used. For immunofluorescence, Alexa Fluor 488, 555 and 647 dyes-conjugated goat anti-rabbit and anti-mouse antibodies (Thermo Fisher Scientific), and donkey anti-mouse FITC and anti-rabbit Cy3 antibodies (Jackson ImmunoResearch) were used.

### Phosphatase and kinase assays

Phosphatase assay was performed on immunoprecipitated AMBRA1 with *lambda* protein phosphatase (λPP) from New England Biolabs and with its specific buffer (1X NEBuffer for PMP, New England Biolabs). λPP activity was inhibited with Halt™ Phosphatase Inhibitor Cocktail (Thermo Fisher Scientific). Reaction mix was incubated for 30 min at 30 °C and protein extracts were denatured and analyzed by WB, as described in WB section.

Kinase assay was performed after MYC-AMBRA1 immunoprecipitation with recombinant CDK1/Cyclin B1 (New England Biolabs or PRECISIO^®^ Kinase from Sigma-Aldrich) and recombinant PLK1 (Millipore or PRECISIO^®^ Kinase from Sigma-Aldrich), in presence of 1X NEBuffer for PK (when using CDK1 from New England Biolabs) or PRECISIO^®^ Kinase Assay Buffer (when using kinases from PRECISIO^®^ Kinase) plus 2 mM phosphatases inhibitors (Sodium fluoride and Sodium orthovanadate) and 10 μCi/mL ^32^P-ATP (Perkin Elmer). Cold kinase assay was performed with 200 μM ATP (Sigma-Aldrich). The two kinases were used alone or in combination and the mix was incubated for 30 min a 30 °C. For combined kinase assay, MYC-AMBRA1 was at first incubated with CDK1 for 30 min and then, after centrifugation for 2 min at 500* g* to discard the old mix, was incubated for other 30 min with PLK1 in presence of 1 μM Roscovitine, to keep CDK1 inactive (modified from [24]). After reaction, protein extracts were denatured and analyzed by autoradiography and WB, as described in the WB section. For autoradiography, nitrocellulose membrane was exposed to film (Amersham) at − 80 °C overnight, and then developed. Background adjustment and cropping of images were done with ImageJ software.

### Filter aided kinase assay for Mass spectrometry analysis

20 µL immunoprecipitated FLAG-AMBRA1 F3 was loaded on a 50 kD MWCO centrifugal Filter (Sartorius, Vivaspin^®^ 500) and washed twice with 200 µL kinase buffer containing 50 mM Tris/HCl pH 7.5, 150 mM NaCl, 2 mM EGTA pH 8.5, 5 mM MgCl_2_, 2 mM DTT. The liquid was removed by centrifugation at 10,000 g for 15 min. On the filter, the sample was incubated with 2 μL CDK1/Cyclin B1 (New England Biolabs) or PLK1 (Millipore) in kinase buffer with 500 µM ATP-Mg^2+^ salt at 30 °C, 900 rpm for 1 h. In the combined kinase experiments samples were first treated with CDK1, which was inhibited after 30 min by adding Roscovitine (Sigma-Aldrich) to final a concentration of 1 µM. Then samples were incubated with PLK1 for another 30 min. The reaction was quenched by adding 200 µL of 8 M Urea within 0.1 M Tris/HCl, pH 8.5. Protein digestion was performed overnight according to the filter-aided sample preparation (FASP) protocol [78]. In order to obtain high sequence coverage, Trypsin and Elastase were used in replicate experiments. On the second day the filter was transferred to a new tube and peptides were eluted twice with 50 µL 50 mM NH_4_HCO_3_. The eluate was acidified with Trifluoroacetic acid (TFA) to final concentration 1% prior Liquid chromatography-Mass spectrometry (LC–MS/MS) analysis.

### Phosphopeptide enrichment for mass spectrometry analysis

Titanium dioxide (GL Sciences Inc., Tokyo, Japan) beads were pretreated with 300 mg/mL Lactic acid in 80% Acetonitrile with 0.1% TFA. The sample was incubated with 2 mg TiO_2_ slurry at room temperature for 30 min. The TiO_2_ beads were spun down and transferred onto a 200 µL tip, which was blocked by a C8 disc (3 M Empore). The tips were washed with 10% Acetonitrile in 0.1% TFA, 80% Acetonitrile in 0.1% TFA and LC–MS grade water. Phosphopeptides were eluted with 50 μl of 1% Ammonia in 20% Acetonitrile and 50 µl of 1% Ammonia in 40% Acetonitrile. The eluate was mixed with 20 μl of 10% Formic acid. The Ammonium formate was removed by vacuum concentration. The dried sample was resuspended with 20 µl of 0.5% Acetic acid for LC–MS/MS analysis. The tip flow-through was stored at − 80 °C for non-phosphopeptide analysis.

### Mass spectrometry analysis

Mass spectrometric (MS) analysis and measurements were performed, as previously described [79], on an LTQ Orbitrap XL Mass Spectrometer (Thermo Fisher Scientific, Bremen, Germany) coupled to an Agilent 1200 or a Q Exactive Plus (Agilent Technologies, Waldbronn, Germany), an nEasy-LC (Thermo Fisher Scientific) or an Eksigent 2D nanoflow-HPLC (AB Sciex, Darmstadt, Germany). HPLC column tips (fused silica) with 75 μm inner diameter (New Objective, Woburn, MA) were self-packed with Reprosil-Pur 120 ODS-3 (Dr. Maisch, Ammerbuch, Germany) to a length of 20 cm. Samples were applied directly onto the column without precolumn. A gradient of A (0.5% Acetic acid in water) and B (0.5% Acetic acid in 80% ACN/water) with increasing organic proportion was used for peptide separation (loading of sample with 2% of solvent B; first separation ramp from 2 to 35% B within 100 min, and second ramp from 35 to 80% B within 20 min).

All raw files were analyzed with the software MaxQuant (Cox and Mann, 2008). Cysteine carbamidomethylation was selected as fixed modification; Methionine oxidation, protein N-terminal acetylation, and phosphorylation on Serine, Threonine, and Tyrosine were selected as variable modifications. In case of SILAC labeled cells, respective labels were also set as quantification mode. A false discovery rate (FDR) of 1% and a minimum length of six amino acids were used for phosphopeptide and site identifications.

All raw files were searched with Mascot 2.2 (http://www.matrixscience.com/). A UniProt Human database version July 2014 containing additional sequences of known typical contaminants (e.g. human Keratins, Trypsin, etc.) was used for searching. For all searches the peptide mass tolerance was ± 10 ppm, minimum Mascot score was 25.

### Flow cytometry

Flow cytometry was performed on cells incubated with Propidium Iodide solution (50 μg/mL Propidium Iodide, 0.1% Triton X-100, 0.1% Sodium citrate, in PBS 1X). After 30 min of incubation at 4 °C and in dark, samples were read at Flow cytometer (FACScan, BD Transduction Laboratories) with red channel (wavelength between 535 and 617 nm) and with a linear scale (FL2-H). 5000–10,000 events were counted for each sample. Data were collected and analyzed with CellQuest software (BD Transduction Laboratories).

### Immunocytochemistry

Cells were grown on poly-Lysine or Fibronectin coated coverslips (Sigma-Aldrich) and immobilized with 4% Paraformaldehyde (Sigma-Aldrich) or 3.7% Formaldehyde (Sigma-Aldrich) plus 30 mM Sucrose (Sigma-Aldrich). Cell permeabilization was performed with 0.1% Triton X-100, then coverslips were blocked with 3% BSA—0.1% Triton X-100 or were quenched with 0.1 M Glycine and blocked with 3% BSA—0.05% Tween-20. For NUMA1 cortical staining cells were washed with Phem Buffer (45 mM Hepes, 45 mM Pipes, 5 mM MgCl_2_, 10 mM EGTA, 1 mM PMSF, pH 6.9), then were incubated with Phem-0,5% Triton X-100, fixed with 4% Paraformaldehyde (Sigma-Aldrich), permeabilized with 0.3% Triton X-100 and then quenched and blocked as described above. Primary antibodies were incubated overnight at 4 °C or for 45 min at RT, and were visualized with the respective fluorescent dyes-conjugated secondary antibodies. Nuclei were stained with 500 ng/mL of Hoechst 33,342 (Thermo Fisher Scientific) or with 0.1 μg/mL DAPI (Sigma-Aldrich).

For mitotic defects analysis, spindle angle measure, and AURKA/pAURKA staining analysis cells were visualized with a Nikon Eclipse 90i microscope, equipped with 100X (oil immersion; N.A. 1.3) objective and a Qicam Fast 1394 CCD camera (QImaging) or with Inverted microscope (Eclipse Ti, Nikon) equipped with 60X (oil immersion, N.A. 1.4) objective and the Clara camera (ANDOR technology). Images were acquired and analyzed with Nis-Elements AR 3.2 (Nikon) or Nis-Elements H.C. 5.11 softwares using the JOBS module for automated acquisitions.

For NUMA1 localization analysis, images were visualized with a Leica confocal microscope (TCS SP8; Leica, Wetzlar, Germany), equipped with a 63X 1.40–0.60 NA HCX Plan Apo oil BL objective at RT, using LasX software for image acquisition and processing.

For PLK1 high-content imaging analysis, cells were grown on fibronectin-coated 96 well microplates (CellCarrier Ultra, Perkin-Elmer) and stained as coverslips. Images were acquired with Operetta imaging system (Perkin-Elmer), and analyzed with Harmony software (Perkin-Elmer).

For P-Ser10 H3 analysis images were visualized with ZEISS AXIO Observer 7 inverted LED microscope (ZEISS), equipped with AXIO cam 360. Images were acquired with ZEN 2.6 software and processed with Fiji software.

### Spindle orientation analysis

For mitotic spindle orientation analysis, cells were fixed and stained as described in the immunocytochemistry section. Only images of cells at metaphase stage were acquired. Mitotic spindle angle was calculated as the angle between the mitotic spindle axis, built using the two centrosomes marked with anti-Pericentrin antibody, and cell growth surface. For mitotic spindle angle measure images were acquired with 100X  or 60X objectives along the z-axis every 0.4 μm for a total range varying from cell to cell, so to include both spindle poles, detected by pericentrin. Angle measure was obtained with mathematical formula α = Arctan(z/xy), where xy is the distance between centrosomes in maximum intensity projection, and z is the distance between the two planes in which centrosomes are in focus along z-axis, as previously described [80].

### Live cell imaging

Time-lapse imaging of cells cultured in 35 mm glass-bottomed dishes (14 mm, No. 1.5, MatTek Corporation) was performed in a heated incubation chamber (37 °C) with controlled humidity and CO_2_ supply (5%), using a Plan-Apochromat DIC 63X/1.4NA oil objective mounted on an inverted Zeiss Axio Observer Z1 microscope (Marianas Imaging Workstation from Intelligent Imaging and Innovations Inc. (3i), Denver, CO, USA), equipped with a CSU-X1 spinning-disk confocal head (Yokogawa Corporation of America) and four laser lines (405 nm, 488 nm, 561 nm and 640 nm). Images were detected using an iXon Ultra 888 EM-CCD camera (Andor Technology).

For quantification of spindle orientation defects and spindle length, HeLa cells were treated with 5 nM SiR-tubulin (Spirochrome) for 1 h prior to imaging. Fifteen 1 µm-separated z-planes were collected every 2 min. The spindle length was quantified by measuring the three-dimensional distance between the spindle poles as described before [81]. Briefly, the distance and number of z-slices between the two spindle poles peak intensities of SiR-tubulin signal were measured using ImageJ (National Institute of Health, Bethesda, MD, USA) and used to quantify the three-dimensional distance between the spindle poles, as well as the corresponding shift in spindle orientation angle. The polar plots of the angles were plotted using a custom written Matlab script (MATLAB R2013b).

Quantification of NUMA1 at the cell cortex was performed as described before [82]. Briefly, a 5 pixel wide line either passing through cortical domains that face each spindle pole at the z-plane with peak spindle pole intensity or along the entire cortex of a sum-projected late prometaphase/metaphase spindle was used to obtain line scan profile and mean fluorescence intensities using ImageJ. The resulting intensity values were normalized to the extracellular and intracellular background intensity quantified using a 20 pixel wide square ROI.

### Spindle elongation analysis

For analysis of spindle elongation during anaphase, HeLa cells stably expressing GFP-NUMA1 were treated with 10 nM SiR-tubulin for 1 h before imaging. Anaphase spindle elongation increase was quantified as the difference in pole-pole distance at 10 min after anaphase onset and metaphase spindle length. Anaphase onset was defined as the time point in which the spindle poles marked by GFP-NUMA1 and SiR-tubulin started to move apart and the time-point before anaphase onset was defined as metaphase. In rescue experiments, quantifications were performed only from cells expressing AMBRA1 identified by mCherry signal.

### Statistical analysis

All the experiments were performed at least three times. For those experiments flanked by a graph, *n* is indicated in figure legends and densitometric analysis was performed using ImageJ software. Histograms show mean ± s.e.m. or s.d., as indicated in figure legends. Statistical significance was assessed using GraphPad Prism software and using ordinary one-way or two-way ANOVA, as indicated in figure legends. For Flow cytometry analysis in supplementary figures, graphs are representative of the experiment shown and were realized with Microsoft Office Excel software. For mitotic spindle angle measure, between 20 and 30 cells for each condition in each experiment were acquired, and the measure of the angle was done by applying the specific formula (see spindle orientation analysis section) with Microsoft Office Excel software. For live cell imaging statistical analysis and graphs were generated in GraphPad Prism and MatLab softwares. Details of statistical tests used and its significance are indicated in the figure legends. The data points were tested for normality using Shapiro–Wilk test. Accordingly, statistical significance was determined by Student’s T test (unpaired, two-tailed) or Mann–Whitney *U* test (unpaired, two-tailed). For parametric tests, the F test was used to compare variances and Welch’s correction was performed accordingly.

### Supplementary Information

Below is the link to the electronic supplementary material.

Supplementary file1 (DOCX 13026 KB)

Supplementary file2 (XLSX 15 KB)

Supplementary file3 (XLSX 18 KB)

Supplementary file4 (AVI 20571 KB)

Supplementary file5 (AVI 20571 KB)

## Data Availability

This study includes the following Datasets: Dataset 1: Phosphosites identified by Mascott Mass Spectrometry on AMBRA1 upon Nocodazole treatment. Dataset 2: Phosphosites identified by Mass Spectrometry on AMBRA1 after in vitro kinase assays using CDK1, PLK1 and the combination of both.

## Abbreviations

AA^1209/1223^: T1209A/S1223A
AMBRA1: Activating molecule in Beclin 1-regulated autophagy
Asyn.: Asynchronous
AURKA: Aurora kinase A
CDK1: Cyclin dependent kinase 1/Cyclin B1
CHX: Cycloheximide
CTR: Control
dTB: Double Thymidine block
F1: Fragment 1
F2: Fragment 2
F3: Fragment 3
F3A: Fragment 3A
F3B: Fragment 3B
FASP: Filter-aided sample preparation
FDR: False discovery rate
FL: Full length
βGal: β-Galactosidase
Interph.: Interphase
LC–MS: Liquid chromatography-Mass spectrometry
KO: Knock-out
LGN: Leu-Gly-Asn repeat-enriched protein
LSB: Laemmli sample buffer
MS: Mass spectrometry
n.s.: Not significant
n.d.: Not detected
Norm.: Normalized
NUMA1: Nuclear mitotic apparatus 1
PBD: Polo-box domain
PEP: Posterior error probability
PIK3C3: Phosphatidylinositol 3-kinase catalytic subunit type 3
PLK1: Polo-like kinase 1
PP: *Lambda* Protein phosphatase
PVDF: Polyvinylidene difluoride
SILAC: Stable isotope labeling with amminoacids in cell culture
TAP: Tandem affinity purification
TFA: Trifluoroacetic acid
ULK1: Unc-51 like kinase-1
WB: Western blot
