# Supplementary material for: AMBRA1 phosphorylation by CDK1 and PLK1 regulates mitotic spindle orientation
Source: Cell Mol Life Sci. 2023 Aug 16;80(9):251. doi: 10.1007/s00018-023-04878-6 (PMC10432340; doi:10.1007/s00018-023-04878-6)

**AMBRA1 phosphorylation by CDK1 and PLK1 regulates**

**mitotic spindle orientation**

Fiorella Faienza,^1,2^ Federica Polverino,^3^ Girish Rajendraprasad,^4^ Giacomo Milletti,^5^ Zehan Hu,^6^ Barbara Colella,^7^ Deborah Gargano,^7^ Flavie Strappazzon,^8,9^ Salvatore Rizza,^10^ Mette Vixø Vistesen,^1^ Yonglun Luo,^11,12^ Manuela Antonioli,^2,13^ Valentina Cianfanelli,^14,15,21^ Caterina Ferraina,^14^ Gian Maria Fimia,^13,16^ Giuseppe Filomeni,^2,10,17^ Daniela De Zio,^18,19^ Joern Dengjel,^6^ Marin Barisic,^4,20^ Giulia Guarguaglini,^3^ Sabrina Di Bartolomeo,^7^* and Francesco Cecconi ^1,22^*

^1^Cell Stress and Survival Unit, Danish Cancer Institute, Copenhagen, Denmark

^2^Department of Biology, University of Rome Tor Vergata, Rome, Italy

^3^Institute of Molecular Biology and Pathology, CNR National Research Council, Rome, Italy

^4^Cell Division and Cytoskeleton, Danish Cancer Institute, Copenhagen, Denmark

^5^DNA Replication and Cancer Group, Danish Cancer Institute, 2100 Copenhagen, Denmark

^6^Department of Biology, University of Fribourg, Fribourg, Switzerland

^7^Department of Biosciences and Territory, University of Molise, Pesche, Italy

^8^IRCCS Fondazione Santa Lucia, Rome, Italy

^9^Univ Lyon, Univ Lyon 1, CNRS, INSERM, Physiopathologie et Génétique du Neurone et du Muscle, UMR5261, U1315, Institut NeuroMyogène, 69008, Lyon, France

^10^Redox Biology Group, Danish Cancer Institute, Copenhagen, Denmark

^11^Lars Bolund Institute of Regenerative Medicine and Qingdao-Europe Advanced Institute for Life Sciences, BGI Research, Shenzhen, China

^12^Department of Biomedicine, Aarhus University, Aarhus, Denmark

^13^National Institute for Infectious Diseases, IRCSS “L. Spallanzani”, Rome, Italy

^14^Department of Pediatric Hemato-Oncology and Cell and Gene Therapy, Bambino Gesù Children's Hospital, IRCCS, Rome, Italy

^15^Department of Science, University "ROMA TRE", 00146 Rome, Italy

^16^Department of Molecular Medicine, Sapienza University of Rome, Rome, Italy

^17^Center for Healthy Aging, University of Copenhagen, Copenhagen, Denmark

^18^Melanoma Research Team, Danish Cancer Institute, Copenhagen, Denmark

^19^Department Of Drug Design And Pharmacology, University Of Copenhagen , Copenhagen, Denmark

^20^Department of Cellular and Molecular Medicine, Faculty of Health Sciences, University of Copenhagen, Copenhagen, Denmark

^21^Department of Woman and Child Health and Public Health, Gynecologic Oncology Unit, Fondazione Policlinico Universitario A. Gemelli IRCCS, Rome, Italy

^22^Institute of Biochemistry and Clinical Biochemistry, Catholic University of the Sacred Heart and IRCCS Policlinico Universitario Fondazione Agostino Gemelli, Rome, Italy

*Correspondence to Francesco Cecconi and Sabrina Di Bartolomeo

**Email:**   [cecconi@cancer.dk](mailto:cecconi@cancer.dk) ; francesco.cecconi@unicatt.it

[sabrina.dibartolomeo@unimol.it](mailto:sabrina.dibartolomeo@unimol.it)

**Supplementary Figure Legends**

**Figure S1. Analysis of synchronization in different cell models.** Cell cycle phase analysis by Flow Cytometry, after suspending cells in Propidium Iodide (PI): **(A)** HeLa cells synchronized at G1/S boundary with a double Thymidine block, and then released in presence of 100 ng/mL Nocodazole. The percentage of cells in each cell cycle phase is shown in the graph on the right. **(B)** Histogram showing quantification of WB in **Fig. 1A** as mean ± s.e.m. of three independent experiments. Significance is n.s. (p > 0.05) by ordinary one-way ANOVA. **(C)** Cell cycle phase analysis by Flow Cytometry, after suspending cells in Propidium Iodide (PI) for HeLa cells synchronized at early mitosis using 200 ng/mL Nocodazole. **(D)** Histogram showing quantification of WB in **Fig. 1B** as mean ± s.e.m. of ten independent experiments. Significance is n.s. (p > 0.05) by Student’s T test. **(E-F)** WB of HCT116 **(E)** and 2FTGH **(F)** cells synchronized at mitosis using 200 ng/mL Nocodazole. The white arrow indicates AMBRA1 electrophoretic migration, while the black arrow indicates its mobility shift. AMBRA1 hypershift was visualized with low percentage acrylamide gels (5-6%)**.** Gel percentages are indicated in each WB panel. **(G)** Cell cycle phase analysis by Flow Cytometry, after suspending cells in Propidium Iodide (PI) for HeLa cells treated with 200 ng/mL Nocodazole and released. The percentage of cells in each cell cycle phase is shown in the graph on the right. Asyn. = “asynchronous.

**Figure S2. CDK1-PLK1 interaction with and phosphorylation of AMBRA1.** **(A)** WB of endogenous proteins immunoprecipitated following Nocodazole treatment with anti-CDK1 antibody. Rabbit immunoglobulins were used as control (IP ctr). **(B)** Phospho-Ser10 H3 staining of HeLa cells treated with Nocodazole and then with 9 μM RO-3306, for CDK1 kinase activity inhibition. The percentage of P Ser10 positive cells is shown in the graph on the right. Scale bar 50 μm. **(C-D)** WB of immunoprecipitated proteins following Nocodazole treatment: **(C)** Endogenous proteins were immunoprecipitated with PLK1 antibody. Mouse immunoglobulins were used as control (IP ctr). **(D)** Overexpressed MYC-AMBRA1 WT or AA^1209/1223^ and MYC-β-Galactosidase as control were immunoprecipitated using anti-MYC antibody. Quantification, only for mitotic protein extracts, as mean ± s.e.m. of three independent experiments is shown, and significance is n.s. (p > 0.05) by Student’s T test. **(E)** Mass spectrometry analysis of an *in vitro* kinase assay with recombinant CDK1/Cyclin B1 and PLK1. F3 FLAG-AMBRA1 was transfected in HeLa cells and then immunoprecipitated with anti-FLAG antibody. Results are presented here as a heat map of phosphopeptide intensities normalized to AMBRA1 protein intensities relative to the CDK1 experiment. Mass spectrometry data are presented in **Dataset 2A** and **2B**. **(F)** WB of an *in vitro* kinase assay with recombinant CDK1/Cyclin B1 and PLK1 performed on MYC-AMBRA1 WT or AA^1209/1223^. Proteins were immunoprecipitated using anti-MYC antibody following HeLa cells transfection with the relative constructs. An asterisk marks a MYC-AMBRA1 degradation sub-product. Gel percentages are indicated in each WB panel.

**Figure S3. *AMBRA1* depletion cause spindle misorientation in HCT-116 and 2FTGH cell lines.** **(A)** WB of HCT-116 cells transfected with siCTR or si*AMBRA1*. **(B)** The same cells as in **A** were used to measure mitotic spindle angle (degrees), that is shown on the left as histogram for all conditions. Bars show mean ± s.e.m. of 50 measures, and significance is **** (p<0.0001) by Mann-Whitney U test. On the right, mitotic spindle angle measure (degrees) is shown as a polar distribution. **(C)** WB of *AMBRA1* KO 2FTGH cells, obtained by the CRISPR/Cas9 technique. **(D)** The same cells as in **C** were used to measure mitotic spindle angle (degrees), that is shown on the left as histogram for all conditions. Bars show mean ± s.e.m. of 20-50 measures, and significance is **** (p<0.0001) by Mann-Whitney U test. On the right, mitotic spindle angle measure (degrees) is shown as a polar distribution. Gel percentages are indicated in each WB panel.

**Figure S4. *AMBRA1* depletion causes defects in mitotic spindle function.** **(A)** WB of CTR and *AMBRA1* KO HeLa cells, obtained by the CRISPR/Cas9 technique. **(B, C)** The same cells as in (**A)** were grown on coverslips and stained with anti-Pericentrin antibody, to identify centrosomes, and anti-α-Tubulin antibody, to mark mitotic spindle. Nuclei were stained with DAPI (scale bar = 10 μm). Single and merged images are shown, with 4X magnification shown only in **C**. White arrows indicate the misaligned chromosomes. The percentage of cells with each defect is represented in the graphs on the right. Bars show mean ± s.e.m. of the percent of cells, which exhibits the indicated defect in three independent experiments. Significance is calculated with Student’s T test: *** (p<0.001). **(D)** *AMBRA1* CTR and KO cells were transfected with WT and AA^1209-1223^ MYC AMBRA1 or with the PLPCX empty vector and were grown on coverslips. Then, they were stained with an anti-MYC antibody, to identify transfected cells, and anti-Pericentrin antibody, to identify centrosomes and build cell division axes. Nuclei were stained with DAPI. Mitotic spindle angle measure (degrees) is shown on the left as histogram for all conditions. Bars show mean ± s.e.m. of 50-80 measures, and significance is **** (p<0.0001) by ordinary one-way ANOVA. On the right, mitotic spindle angle measure (degrees) is shown as a polar distribution. **(E)** Live cell imaging of CTR and *AMBRA1* KO HeLa, stained with SiR-tubulin. On the left are shown representative images of CTR and *AMBRA1* KO cells exhibiting spindle positioning defects. Single z-slice with an orthogonal projection is shown. Scale bar: 10µm (xy), 2µm (xz and yz). Spindle angle deviation is shown as polar distribution plots on the right. N(number of cells, number of independent experiments): CTR (39, 3); *AMBRA1* KO (47, 3). **(F)** Graph depicting spindle length quantified as pole-pole distance from **E**. Individual spindles along with mean ± s.d. is plotted. P-values were calculated using Mann-Whitney U test: n.s. (p > 0,05). **(G)** WB of HeLa DHC-GFP cells transfected with siCTR or si*AMBRA1*. **(H)** The same cells as in **G** were analyzed by confocal live-cell imaging microscopy and the spindle angle was measured. Measures are shown as polar distribution plots. N(number of cells, number of independent experiments): siCTR (41, 3); si*AMBRA1* (36, 3). Gel percentages are indicated in each WB panel.

**Figure S5. AMBRA1 phosphorylation has no effect on autophagy regulation.** **(A-B)** WB of stable *AMBRA1*-silenced Hek293 cells transfected with WT and phosphosilent (AA^1209/1223^) MYC-AMBRA1 or with the PLPCX empty vector. Cells were synchronized at mitosis using 100 µM Monastrol and in B were also treated or not with 20 μM Chloroquine (CQ). Protein quantification, as the mean ± s.e.m. of three independent experiments, is shown in the graphs on the left for **A** and at the bottom for **B**. Significance is n.s. (p > 0.05) by two-way ANOVA. Gel percentages are indicated in each WB panel.

**Figure S6. Analysis of NUMA1 and AMBRA1 localization in mitotic cells.** **(A)** Live cell imaging of CTR and *AMBRA1* KO HeLa cells, obtained by the CRISPR/Cas9 technique and transfected with GFP-NUMA1. A single z-slice of the spindle pole axis and localization of GFP-NUMA1 at the cortex is shown along with the microtubule spindle stained with SiR-tubulin. Scale bar: 10 µm. **(B)** The same cells as in **A** were used to calculate GFP-NUMA1 signal intensity across the whole cortex in the indicated cell lines. On the top is an illustration showing how GFP-NUMA1 intensity was calculated. On the bottom is the graph with NUMA1 signal intensity with mean ± s.d. P-values were calculated using Mann-Whitney U test and significance is: **** (p<0.0001). N(number of cells, number of independent experiments): siCTR (33, 3); si*AMBRA1* (31, 3). **(C)** The same cells as in **A** were used to calculate GFP-NUMA1 levels at the cortex extracted from line profiles. On the top is an illustration showing the line scan for GFP-NUMA1 cortical intensity calculation. On the bottom are NUMA1 levels at the cortex extracted from line profiles. Individual cortical intensities with mean ± s.d. are plotted. P-values were calculated using Mann-Whitney U test and significance is: **** (p<0.0001). N(number of cells, number of independent experiments): CTR (29, 3); *AMBRA1* KO (30, 3). On the right are shown NUMA1 line profiles across the cortex. The solid line represents the mean intensity of measures with s.d. (dotted lines). **(D)** NUMA1 signal intensity for stable *AMBRA1*-silenced HeLa cells. NUMA1 signal intensity was normalized over α-TUBULIN signal intensities and is shown as mean ± s.e.m. of about 100 measures. Significance is **** (p<0.0001) by Student’s T. **(E)** Representative spinning-disk confocal images showing the localization of mCherry-AMBRA1 WT and mCherry-AMBRA1 AA^1209/1223^ mutant during metaphase. HeLa cells were transiently transfected with corresponding plasmids and microtubules stained using SiR-tubulin. Scale bar: 10 µm. Relative films are shown in **Movie 1** (WT AMBRA1) and **Movie 2** (AA^1209/1223^ AMBRA1).

**Figure S7. Analysis of different markers in *AMBRA1*-silenced and rescued cells.** **(A-D)** WB of stable *AMBRA1*-silenced HeLa cells transfected with WT and phosphosilent (AA^1209/1223^) MYC-AMBRA1 or with the PLPCX empty vector and synchronized at mitosis with Nocodazole. Protein quantification as the mean ± s.e.m. of four **(A, B)** or three **(C, D)** independent experiments, is shown in the graphs on the bottom. Significance is n.s. (p > 0.05) by one-way ANOVA. **(E)** WB of immunoprecipitated proteins following Nocodazole treatment. Overexpressed MYC-AMBRA1 WT or AA^1209/1223^ and MYC-β-Galactosidase as control are immunoprecipitated using anti-MYC antibody. Quantification, only for mitotic protein extracts, as mean ± s.e.m. three independent experiments, is shown on the right, and significance is n.s. (p>0.05) by Student’s T test. **(.F)** WB analysis of protein extracts from CTR and *AMBRA1* KO HeLa cells treated with: **(E)** 100 μg/mL of cycloheximide (CHX) for the indicated time points alone or in combination with 5 μM MG132. Quantification, as mean ± s.e.m. three independent experiments, is shown on the right and significance is n.s. (p>0.05), * (p < 0.05), and ** (p < 0.01) by ordinary two-way ANOVA. **(G, H)** WB of stable NUMA1-GFP HeLa cells transfected with si*AMBRA1*, si*AMBRA1* (5’UTR) **(G)** or with si*AMBRA1* (5’UTR) and rescued with mCherry-AMBRA1 WT, AA^1209/1223^, and PXP **(H)**. . An asterisk marks a MYC-AMBRA1 degradation sub-product. Gel percentages are indicated in each WB panel.

**Movies**

**Movie 1.** Live cell imaging of mCherry-AMBRA1 WT localization in mitotic cells.

**Movie 2.** Live cell imaging of mCherry-AMBRA1 AA^1209/1223^ localization in mitotic cells.


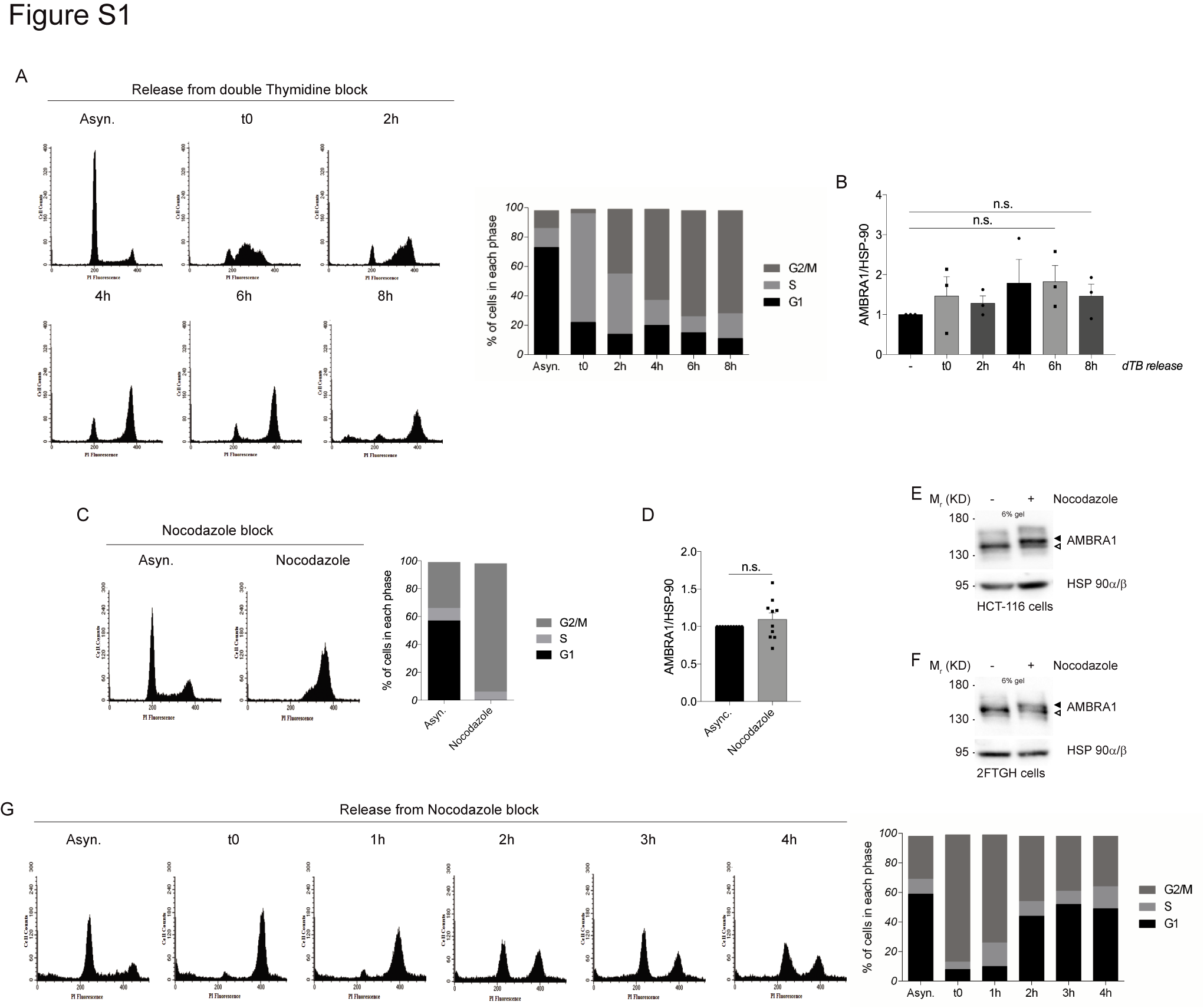


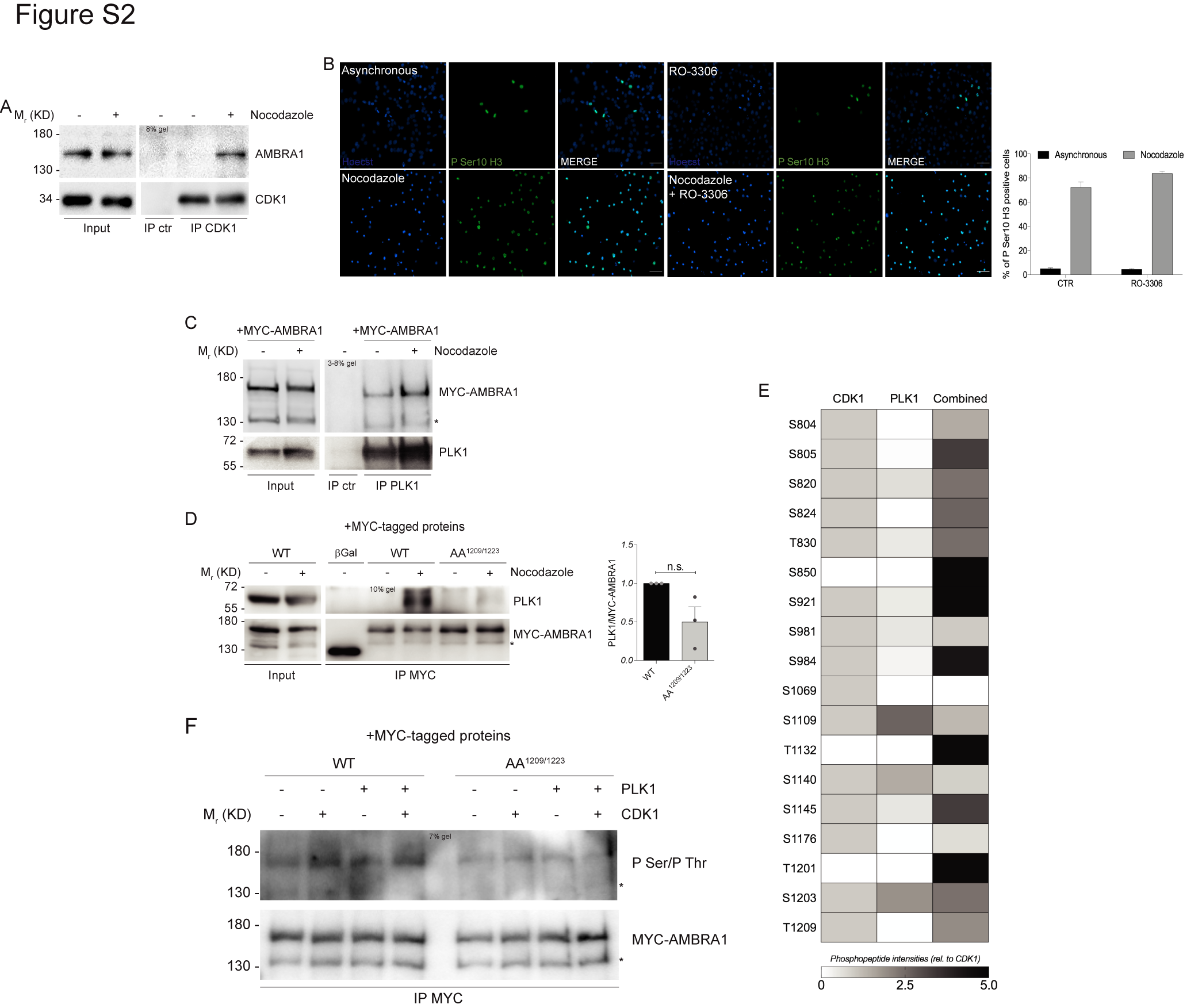


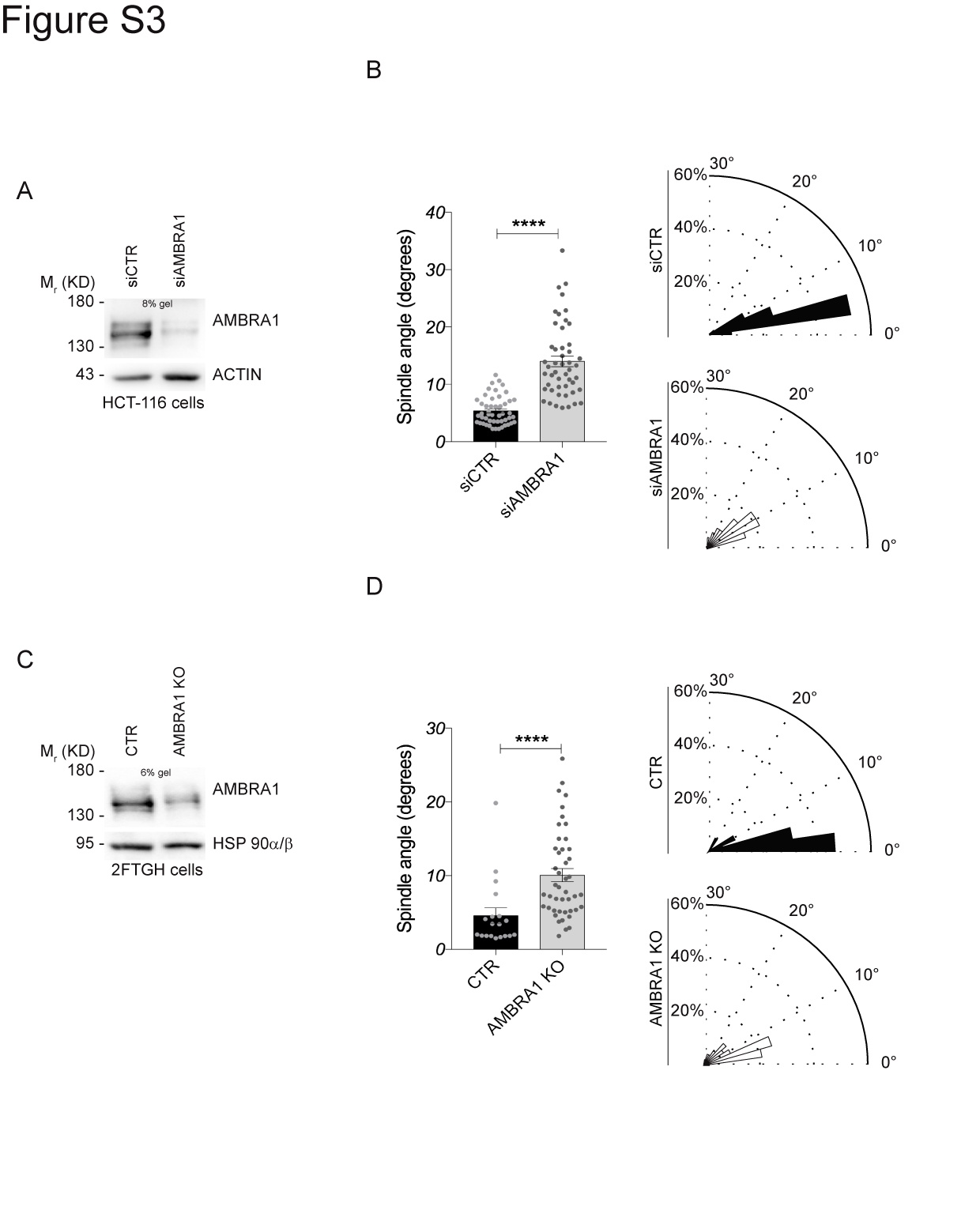


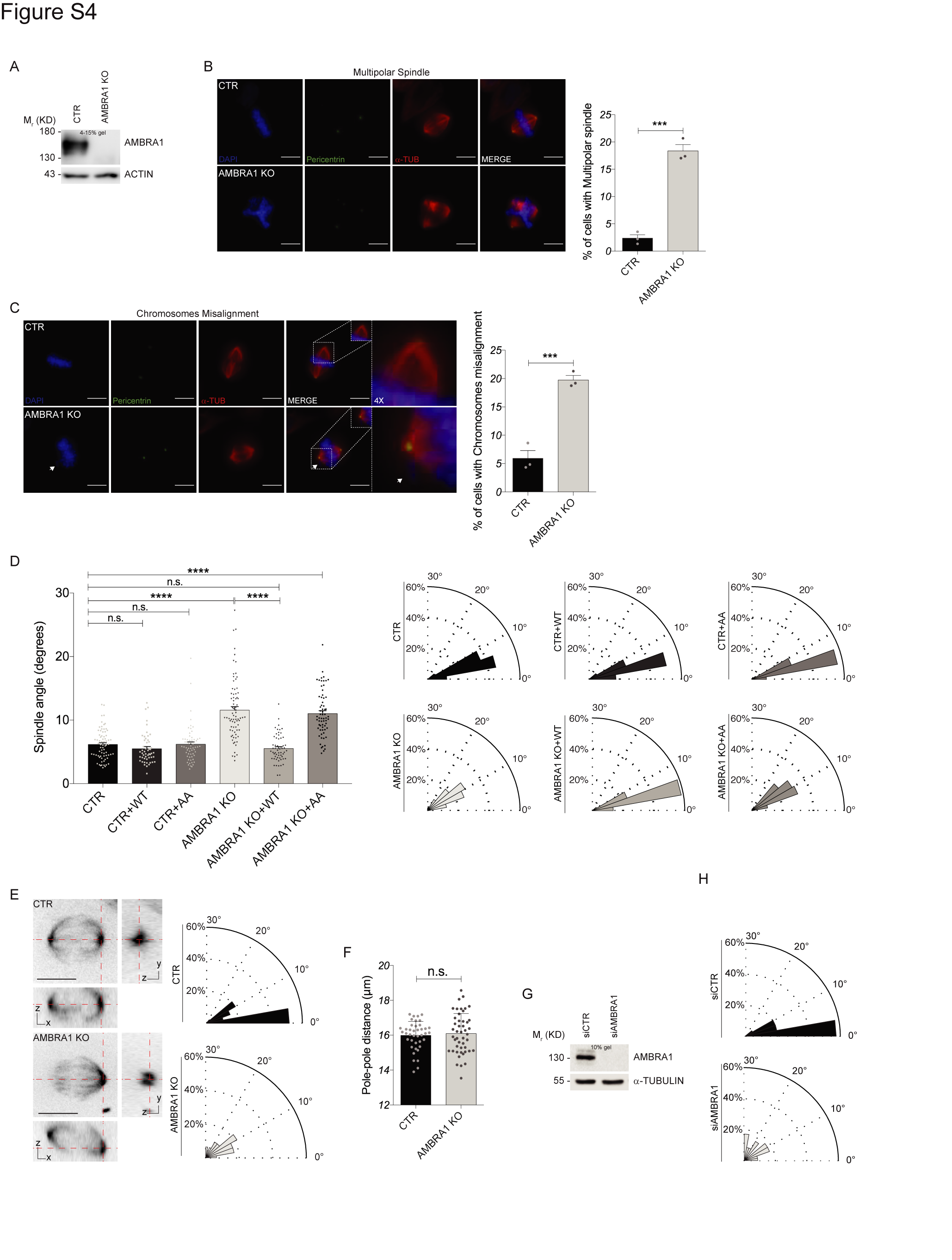


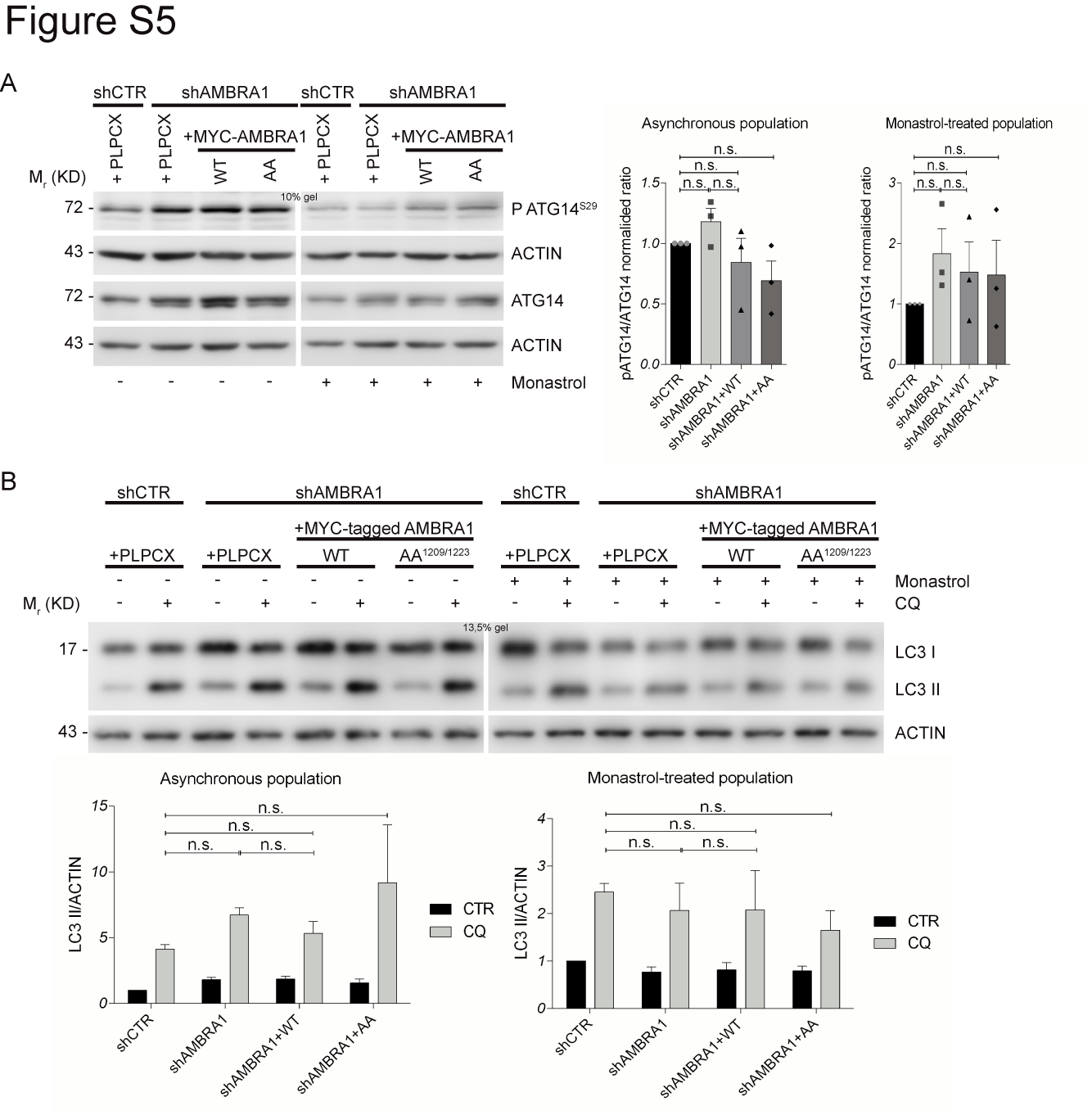


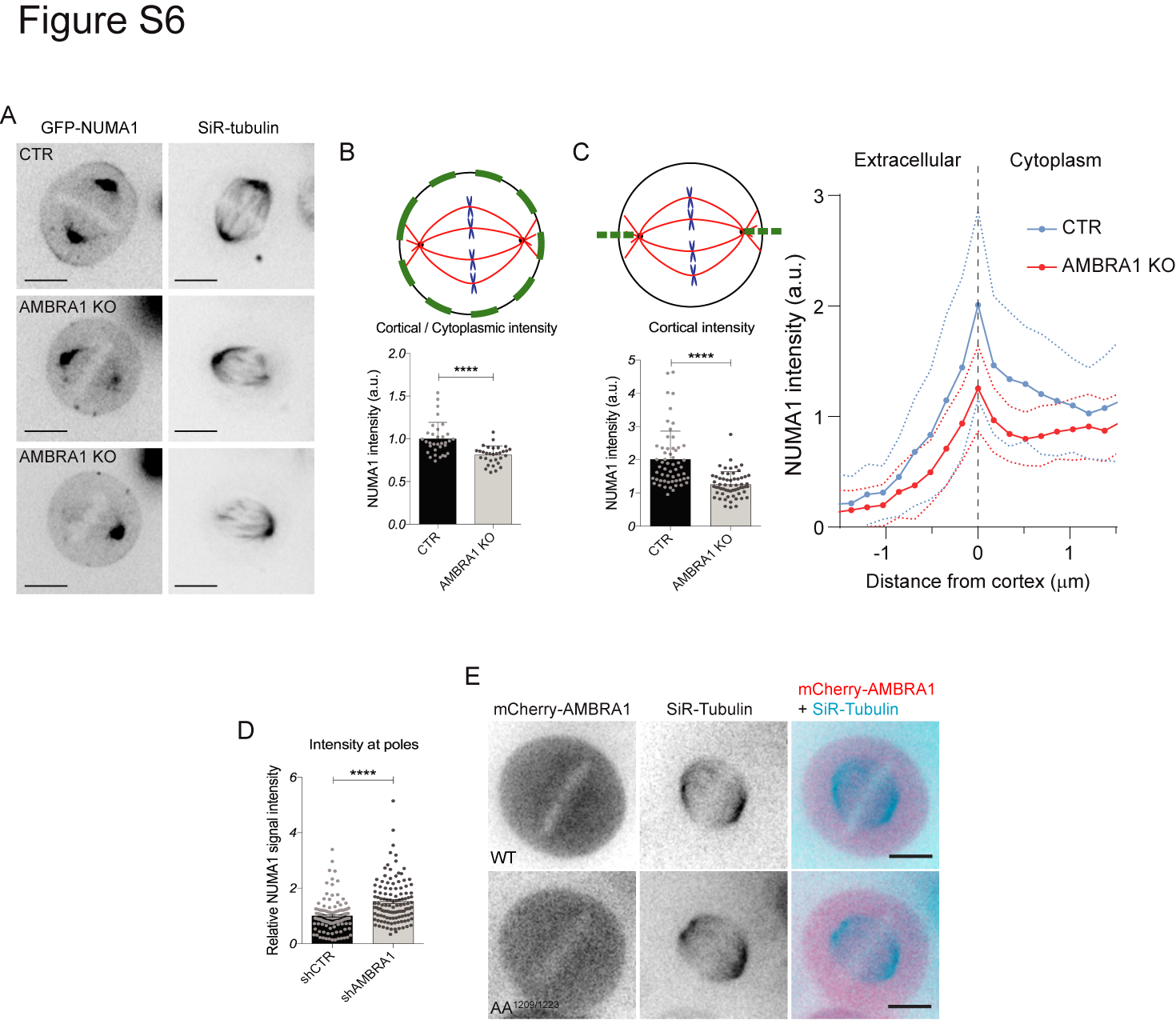


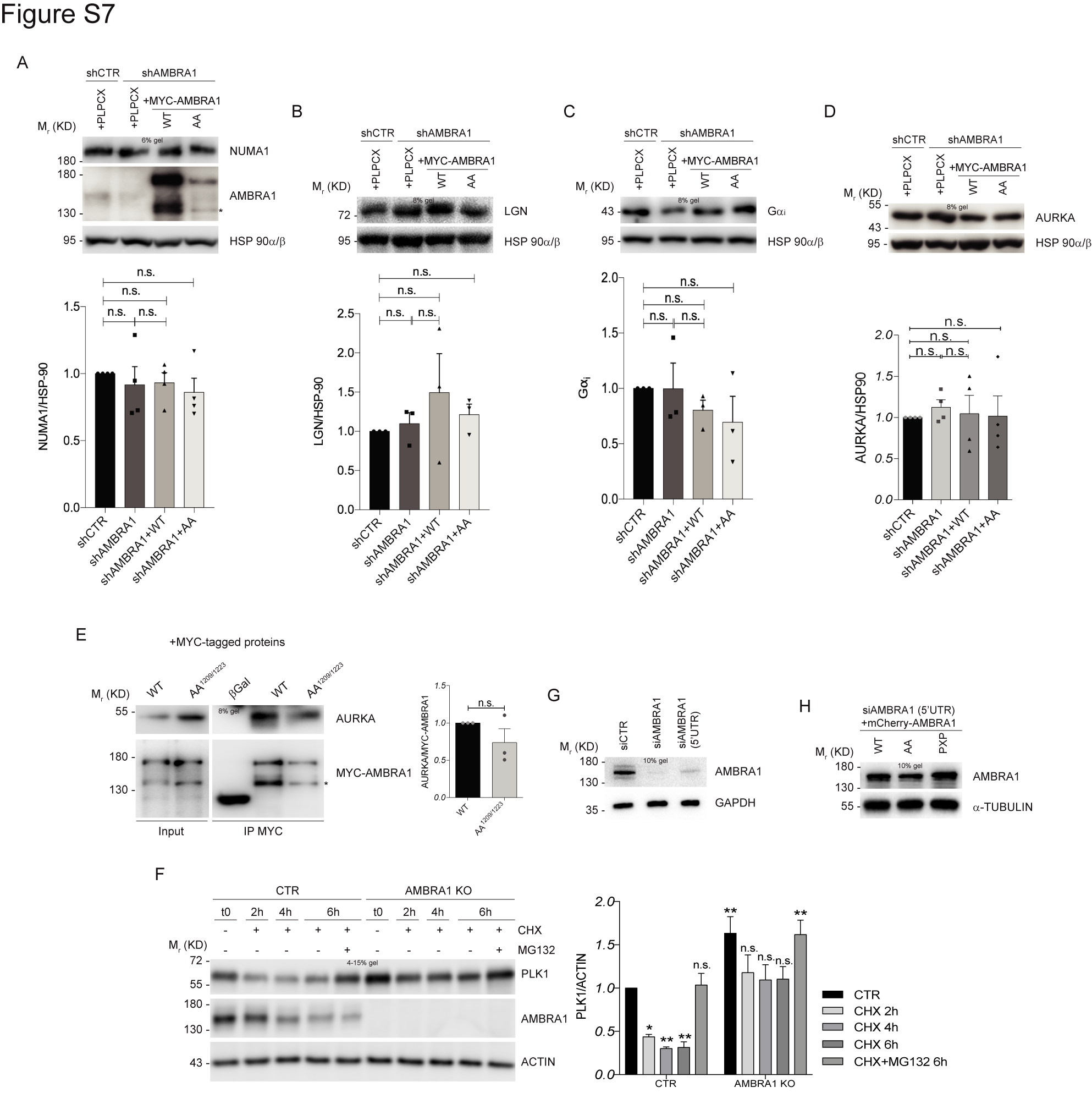

Supplement: Supplementary file 1 — Supplementary file1 (DOCX 13026 KB) [file 18_2023_4878_MOESM1_ESM.docx]
